# Pyroglutamyl-N-terminal prion protein fragments in sheep brain following the development of transmissible spongiform encephalopathies

**DOI:** 10.3389/fmolb.2015.00007

**Published:** 2015-03-11

**Authors:** Adriana Gielbert, Jemma K. Thorne, James Hope

**Affiliations:** ^1^Department of Pathology, Animal and Plant Health Agency-WeybridgeAddlestone, UK; ^2^Science Strategy Group, Animal and Plant Health Agency-WeybridgeAddlestone, UK

**Keywords:** pyroglutamate, amyloid disease, prions, mass spectrometry, strain differentiation

## Abstract

Protein misfolding, protein aggregation and disruption to cellular proteostasis are key processes in the propagation of disease and, in some progressive neurodegenerative diseases of the central nervous system, the misfolded protein can act as a self-replicating template or prion converting its normal isoform into a misfolded copy of itself. We have investigated the sheep transmissible spongiform encephalopathy, scrapie, and developed a multiple selected reaction monitoring (mSRM) mass spectrometry assay to quantify brain peptides representing the “ragged” N-terminus and the core of ovine prion protein (PrP^Sc^) by using Q-Tof mass spectrometry. This allowed us to identify pyroglutamylated N-terminal fragments of PrP^Sc^ at residues 86, 95 and 101, and establish that these fragments were likely to be the result of *in vivo* processes. We found that the ratios of pyroglutamylated PrP^Sc^ fragments were different in sheep of different breeds and geographical origin, and our expanded ovine PrP^Sc^ assay was able to determine the ratio and allotypes of PrP accumulating in diseased brain of PrP heterozygous sheep; it also revealed significant differences between N-terminal amino acid profiles (N-TAAPs) in other types of ovine prion disease, CH1641 scrapie and ovine BSE. Variable rates of PrP misfolding, aggregation and degradation are the likely basis for phenotypic (or strain) differences in prion-affected animals and our mass spectrometry-based approach allows the simultaneous investigation of factors such as post-translational modification (pyroglutamyl formation), conformation (by N-TAAP analysis) and amino-acid polymorphisms (allotype ratio) which affect the kinetics of these proteostatic processes.

## Introduction

The mammalian prion protein (PrP^C^) is a di-N-glycosylated, glycophosphatidyl–inositol (GPI-) tailed membrane protein of approximately 220–250 amino acids. In mammals of several genera, aberrant isoforms of prion protein (PrP^Sc^) are associated with the class of neurodegenerative diseases called transmissible spongiform encephalopathies (TSEs), or prion diseases. TSEs can have different incubation times, rates of progression, distributions of the PrP^Sc^ aggregates at the level of the whole organism, tissue and cell, and differences in the protease resistance of PrP^Sc^. Variable rates of PrP misfolding, aggregation, and degradation are the likely basis for phenotypic (or strain) differences in prion-affected animals. For example, small differences in the amino-acid sequence of PrP^C^ are known to be associated with variations in disease manifestation, which is particularly evident in inherited forms of the disease in humans (Kovacs et al., 2005). Similarly, conformational differences (detected by variable N-terminal proteolytic cleavage of PrP^Sc^) or post-translational modification (the number of N-glycans) have been identified as crude molecular indicators of biological type and potency.

Scrapie is the TSE of sheep and goats and the susceptibility and relative survival time of these small ruminants affected by scrapie are associated with the amino-acid sequence of their prion protein. In ovine PrP, there are three common polymorphisms of ovPrP in the domestic sheep population—at codon 136, valine (V) or alanine (A); at codon 154, histidine (H) or arginine (R); and at codon 171, glutamine (Q), arginine (R), or histidine (H). These are found in five most abundant combinations or alleles VRQ, ARQ, ARH, AHQ, and ARR, and this ranking of alleles mirrors their decreasing susceptibility to classical forms of scrapie when found in homozygous genotypes. Scrapie can manifest itself as distinct isolates or strains with characteristic and reproducible phenotypes (Bruce, 1993), and these sheep phenotypes have been crudely classified by molecular typing of the protease-resistant core of PrP^Sc^ by Western blotting (Hope et al., 1999; Everest et al., 2006). A more detailed knowledge of the tertiary and quaternary structure of PrP^Sc^ would be useful as an epidemiological tool to map the source and spread of TSEs in a population, with the same precision as can be currently inferred from RNA or DNA sequencing of viral and bacterial isolate genomes.

We have previously reported the use of multiple selected reaction monitoring (mSRM) mass spectrometry to quantify peptides representing a number of specific sites of the “ragged” N-terminus of PrP^res^, obtained by treatment of sheep brain tissue homogenate with proteinase K (PK). Four TSE isolate types [CH1641, SSBP/1, the cattle TSE, bovine spongiform encephalopathy (BSE), and one type of classical scrapie] were characterized by this method, termed N-terminal amino acid profiling (N-TAAP) (Gielbert et al., 2009). The relative abundance (RA) of these N-terminal cleavages was found to allow differentiation between TSEs. Subsequently we applied this method as an epidemiological tool to identify TSE strains and subtypes from a set of 29 naturally infected scrapie cases (Gielbert et al., 2013). Our previous mSRM-based assay for ovine TSE differentiation included 7 semi-tryptic peptides with N-termini G77, G81, G85, G89, G94, G96, and W102 and C-terminus K109, of the ovine prion protein representing seven potential PK cleavage sites of PrP^Sc^ (Gielbert et al., 2013). Since, for a number of PrP^res^ preparations, the ratio of total N-TAAP peptide to core- and C-terminal tryptic peptide concentrations was considerably different from unity, we investigated whether more N-terminal cleavage sites might exist than were represented in the assay. This led us to identify pyroglutamyl-N-terminal PrP peptides in scrapie-infected sheep brain and here we describe our further investigation of peptides with this post-translational modification.

While pyroglutamate (pE) formation can occur as an artifact resulting from spontaneous conversion of N-terminal glutamyl residues in weak acidic conditions and is a well-known phenomenon in biological mass spectrometry (Carr and Biemann, 1984), it can also occur as the result of a biological conversion by glutamyl cyclase (Mori et al., 1992; Garden et al., 1999; Cynis et al., 2006). This has been demonstrated for a number of proteins and peptides (Mandal and Balaram, 2007), for instance in recombinant monoclonal antibodies (Chelius et al., 2006), and is of particular interest in relation to amyloid formation (Momoi et al., 1995; Schilling et al., 2006, 2008; Manuelidis, 2007; Cynis et al., 2008; Schlenzig et al., 2009; Jawhar et al., 2011a; Nussbaum et al., 2012; Saul et al., 2013). Intriguingly, N-terminally truncated and pE-amyloid β (Aβ) peptides have been identified by mass spectrometry as constituents of amyloid deposits in Alzheimer's disease (Mori et al., 1992; Portelius et al., 2010) and postulated to have “prion-like” properties (Nussbaum et al., 2012; Matos et al., 2014). Similarly, PrP^Sc^ fragments with N-terminal pE may be formed *in vivo* and might be directly related to the prion behavior of PrP^Sc^, while it is also possible that pyroglutamyl PrP peptides are artifacts resulting from sample preparation.

To establish whether this is an artifact or if such modifications are a result of *in vivo* processes, we determined the rates of pE formation under typical sample processing conditions. Knowing the kinetics of *in vitro* pE formation allowed us to establish a work flow for the detection of pE modifications in PrP^Sc^ obtained from TSE samples before *in vitro* modification obscures these data. We also expanded our ovine PrP peptide assay to include N-TAAP peptides starting with any residue from G85, and many tryptic peptides uncovered from both tissue and recombinant PrP full-scan analyses. To define the increased discriminatory power of this expanded assay in the study of ovine TSEs, we applied the methodology to determine peptide profiles in ovine TSE samples of various origins and report these data herein.

## Materials and methods

### CNS samples from TSE-affected sheep

All samples were prepared and processed in our laboratories at UK Advisory Committee for Dangerous Pathogens (ACDP) Containment Level 3 with derogation for TSEs. Frozen brain tissue samples from sheep infected with TSEs were obtained from the Animal and Plant Health Agency (AHPA) Biological Archive. Natural classical scrapie (pons) samples originated from a flock of mixed breed and genotype sheep at the AHPA in which scrapie was endemic. There are potentially multiple scrapie types in this flock given that it is populated by sheep exposed to scrapie-affected animals from various sources imported into the flock over several years (Ryder et al., 2004). Sheep in this flock were either home bred or purchased into the flock. Brain stem (thalamus) samples were obtained from sheep (neutered male Cheviot, AHQ/AHQ genotype) experimentally challenged with BSE (5 g oral dose) while experimental CH1641 scrapie samples (frontal cortex) were obtained from neutered male Cheviot, AHQ/AHQ genotype, sheep (Gielbert et al., 2009).

All TSE-affected animals were euthanized following onset of clinical signs of disease. Brain samples were collected post-mortem, snap-frozen within 1 h and stored at −80°C until required. A diagnosis of TSE was confirmed by histo-pathological examination of brain sections, and detection of abnormal prion protein by immunohistochemistry and Western blotting, prior to N-TAAP profiling by mass spectrometry.

All procedures involving animals were approved by the Home Office under the UK Animal (Scientific Procedures) Act 1986, and following internal ethical review processes as mandated by the Home Office.

### Preparation of PrP^res^ and tryptic peptides

All chemicals were of reagent grade or better and obtained from Sigma-Aldrich unless otherwise stated. Brain tissues for Q-Tof analysis (classical scrapie: 2.2 g brain stem from a VRQ/VRQ genotype animal; experimental BSE: 0.5 g brain stem from an ARQ/ARQ animal; CH1641 scrapie: 1.2 g cortex from an AHQ/AHQ animal; 1.5 g brain stem from an ARQ/ARQ control) were each homogenized in 16 ml 0.01 M PBS pH 7.4 containing 10% N-lauroyl sarcosinate, by mechanical shearing for 60 s, using an Omni GLH homogenizer (Omni International). Brain tissues for chip-HPLC mSRM QQQ analysis (300 ± 50 mg) were homogenized to 20% in 0.01 M PBS pH 7.4 containing protease inhibitor (cOmplete ULTRA tablets, Mini, EDTA free, Roche Diagnostics) by grinding in a sample homogenizer (Bio-Rad) at 6.5 Hz for two 45 s cycles. Subsequently, homogenates were diluted to 10% by addition of 10% N-lauroyl sarcosinate in 0.01 M PBS pH 7.4. The 10% homogenates were first centrifuged at 17,000 g for 2 min; subsequently the supernatant was selected and centrifuged at 338,000 g for 30 min. Resulting pellets were suspended in water (200 μl) to which 15% KI in 0.01 M Tris-HCl (pH 7.4) containing 1.5% sodium thiosulfate and 1% N-lauroyl sarcosinate (400 μl) was added. At this point, suspensions were divided into 2 × 300 μl aliquots which were processed and analyzed in parallel. To each 300 μl aliquot, 6 μl Proteinase K solution (39 units/mg, 2 mg/ml in water) was added. Following incubation at 37°C for 30 min under agitation (Thermoshaker, 1000 rpm), 6 μl Pefablock solution (6 mg/ml) was added to stop the reaction.

This solution containing PrP^res^ was processed further, either (preparations for analysis by chip-HPLC full-scan Q-Tof) by careful transfer of the solution onto a cushion of 20% sucrose in iodide solution (0.9 M potassium iodide, 9 mM sodium thiosulphate, 15 mM sodium phosphate, pH8 and 1% N-lauroyl sarcosinate, 0.6 ml) in a 5 ml tube, topped up with iodide solution and centrifuged at 176,000 g for 30 min at 10°C, or (preparations for analysis by chip-HPLC mSRM QQQ) by addition of 300 μl of a 1-propanol/1-butanol (1/1) mixture, following which suspensions were vortex-mixed and centrifuged at 24,000 g for 30 min. Pellets were suspended in water (100 μl), 1 M NaCl was added (900 μl), then suspensions were vortex-mixed and centrifuged for 10 min at 15,000 g.

Following solubilization of the pellets in guanidine hydrochloride (GuHCl, 100 μl, 6M in 50 mM Tris, pH 8.0), PrP^res^ in the pellets was reduced with 2mM 1,4-dithioerythritol at 95°C for 20 min and alkylated with 4-vinylpyridine (10% in water, 1 μl) for 10 min at ambient temperature. Insoluble material was discarded following centrifugation (2 min, 11,000 g) and protein was isolated from the supernatant following precipitation with cold methanol (900 μl, −20°C) maintaining −20°C overnight before centrifugation at 10,000 g for 10 min at −4°C. The supernatant was discarded and the pellet resuspended in cold methanol (100 μl, −20°C), centrifuged at 10,000 g for 2 min at −4°C and after discarding of the supernatant the pellet was allowed to dry at ambient temperature for 20 min. The pellet was suspended in 10 μl freshly prepared urea (9M). Sample preparations for analysis by chip-HPLC mSRM QQQ underwent ultrasonication (60 s at level 6, Misonix XL 2020 with cuphorn adaptor and cooler, Misonix, Farmingdale, NY) to assist solubilization at this stage and following the earlier solubilization in GuHCl. Subsequently 10 μl of a buffer consisting of 150 mM Trizma base, 60 mM methylamine-HCl and 15 mM calcium acetated adjusted to pH 8.3 with glacial acetic acid, and 2 μl of the synthetic trypsin substrate boc-vla-leu-lys-7-amido-4-methylcoumarin (1 ng/μl) were added. Trypsin (sequencing grade, Promega) was dissolved in buffer in accordance with the manufacturer's instructions (100 ng/μl,) and 3μl was added to each PrP^res^ preparation. Following trypsin digestion at 30°C for 18 h, the reaction was terminated by addition of 12 μl 5% v/v formic acid (FA). Thus, each 300 mg sample of brain gave rise to two replicates of PK-treated and trypsin digested PrP^res^ preparations. Digests that could not be analyzed by chip-HPLC mSRM within 18 h of this final preparation step (see Results) were stored at −20°C until analysis. As a control and to allow determination of tryptic peptides without complications resulting from truncation or glycosylation, 360 ng (16 pmol) recombinant ovine PrP (136A, L141 154R, 171Q) was dissolved in water (50 μl), precipitated using nine volumes of methanol, and reduced, alkylated and digested with trypsin using the same quantities of reagent as above.

### Chip-HPLC and mass spectrometry

Full scan mass spectrometry analyses were carried out using an Agilent 6520 Q-Tof mass spectrometer interfaced with a Chip Cube and Agilent 1200 nano-HPLC system (Agilent, UK). PrP^res^ tryptic digest samples were injected (1 μl) onto an Agilent Ultra-high Capacity chip, containing a 500 nl enrichment column and a 150 mm × 75μm analytical column custom packed with ACE AQ, particle size 5 μm (UHC-ACE AQ chip).

For N-terminal glutamine conversion rate determinations, custom synthesized peptide standards with N-terminal glutamine or pyroglutamate residues (min. 98% purity; Peptide Protein Research Ltd., Eastleigh, UK) were diluted into post-trypsin buffer (PTB, 5M urea in 25 mM Tris-methylamine pH 8.3) to which half a volume 5% FA in water (PTB-FA), half a volume 5% HCl in water (PTB-HCl), half a volume water (PTB-water), or just water, to a final concentration of 100 fmol/μl. Peptide solutions thus prepared were placed in the HPLC autosampler compartment set to 8°C, immediately injected (1 μl) onto an Agilent ProtID chip with a 40 nl enrichment column and a 150 mm × 75μm analytical column packed with Zorbax 300SB-C18 material. A HPLC-MS analysis sequence initially consisting of eight cycles of analyses of N-terminal glutamyl or pyroglutamyl peptide mixtures in water, in PTB-FA and in PTB-HCl, spaced by buffer blanks, was executed and the injection times of individual analyses recorded; the total duration of the sequence was approximately 20 h. Sequences consisting of four cycles each were subsequently executed 144, 216, 288, and 384 h after the start of the original sequence and peptide mixtures in PBT-water were included in the sequences from 216 h onwards. For sample loading, a continuous flow (4 μl/min) of 0.1% TFA in water (LC-MS grade, Fisher Scientific) was used and the chip injection flush volume set to 8 μl. For analysis, a gradient was used running from 3 to 40% solvent B [where solvent A was 0.1% v/v FA in water and solvent B was 0.1% FA in 90% acetonitrile (LC-MS grade) and 10% water] over 20 min at a flow rate of 500 nl/min. The capillary voltage was set to 1900 V. Both MS/MS and MS-only spectra were acquired, in centroid mode over the mass range of 250–3000 u and at a scan rate of 1 spectrum/s, with the MS abs. threshold set to 200 and the MS rel. threshold to 0.010%. The Agilent ESI calibration standard at m/z 922.009798 was used as internal mass reference.

**Figure 1:**
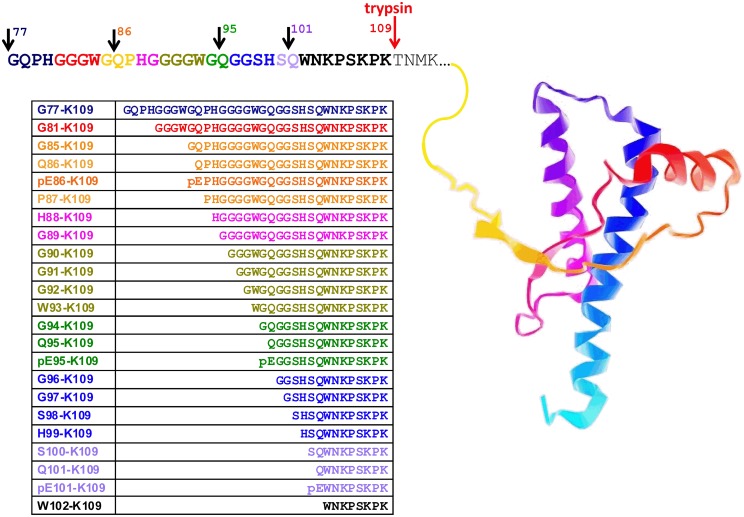
**Ovine prion protein model showing the amino acid sequence in the partially protease-sensitive region**. Selected PK sites and the tryptic cleavage site are pointed out by the arrows. The resulting N-TAAP peptides and their N-terminal pyroglutamyl analogs where applicable are shown in the accompanying table, together with the sequence references used. The ovine PrP structure shown is for illustration only and was captured from Protein Data Bank entry 1Y2S (ovine PrP fragment V124-S234) using GLmol – Molecular Viewer (Version 0.47).

Analogs of other peptides representing relevant sequences of ovine PrP (Figure 1) were also custom synthesized (min. 98% purity; Peptide Protein Research Ltd) and used, without further purification, for method optimization and as external calibration standards for quantification. A multiplexed LC-MS/MS method was developed using an Agilent 6410 triple quadrupole mass spectrometer interfaced with a Chip Cube and Agilent 1200 nano-HPLC system (Agilent, UK). A dynamic mSRM method was developed based on synthetic versions of each of the new peptides to be included in the expanded ovine PrP assay; MS/MS data were acquired for each peptide scanning an appropriate mass range, and two to four precursor/product ion pairs (transitions) selected and optimized (Table S3).

Peptide calibration standards or tryptic digest samples were injected (1 μl) onto an Agilent Ultra-high Capacity chip, containing a 500 nl enrichment column and a 150 mm × 75μm analytical column packed with Zorbax 80-SB-C18, particle size 5 μm. For the loading pump 0.1% FA in water (LC-MS grade, Fisher Scientific) was used (3 μl/min). The chip injection flush volume was set to 8 μl. Analyte separation and elution into the mass spectrometer was carried out in forward flush mode. A gradient was used running from 3 to 50% solvent B [where solvent A was 0.1% v/v FA in water and solvent B was 0.1% FA in 90% acetonitrile and 10% water (LC-MS grade, Fisher Scientific) over 25 min at a flow rate of 400 nl/min]. The capillary voltage was set to 1900 V.

Retention times were determined using the PrP peptide standards and retention time windows set at 2 min for most peptides; for peptides which tended to have wider peaks or peaks that tended to shift as the assay progressed, up to 4 min were used.

Dilution series of calibration and quality control (QC) standards ranging between 0.02 fmol/μl and 1 pmol/μl were run at the start of the sample batch; additional QC standards at the end and, depending on the number of preparations analyzed, mid-batch. To minimize carryover effects, duplicate injections of 8 μl 70/30 (v/v) acetonitrile/water followed by two or more blank runs were inserted after the highest concentration standards and between preparations from different samples. The limit of detection (LoD) was defined as the concentration above which peptides could be detected with a signal to noise ratio larger than 3, or with a signal exceeding that in the second blank used to minimize carryover effects, by a factor of 3, whichever was higher. The lower limit of quantification (LloQ) was defined as the concentration above which the mean concentration determined for three QC standards was within 20% of the expected concentration (Table S4).

### Data processing, analysis, and calculations

The BioConfirm module in the Agilent Qualitative Analysis software (B.06.00) was used to deconvolute MS-only spectra using the “Find by Molecular Feature” method. Here, the “Small Molecules (chromatographic)” extraction algorithm was used, the input data range restricted to 250–2000 Da, peaks accepted with heights ≥10 counts, H^+^, Na^+^, and K^+^ ions included, isotope grouping peak spacing tolerance set to 0.0025 m/z plus 7 ppm, isotope model set to “peptides,” assigned charge states limit set to 15 and compound quality score ≥80. No other compound filters, mass filters or mass defect filtering were selected. The resulting candidate compounds were then interrogated for semi-tryptic (*n* = 51) and tryptic (*n* = 100) PrP peptides (see Tables S1, S2) using custom-defined protein digests of PrP fragments created within the “Define and Match Sequences” method such that R-P cleavages, missed cleavages and variations resulting from polymorphisms A/V136, L/F141, H/R151, and Q/H/R171 were taken into account. The software option to include frequently encountered post-translational modifications such as oxidation, deamidation and decarbamylation was selected. MS match tolerance was set to 10 ppm and MS score to 30. The results were carefully inspected, evaluated and the most likely peptide candidates selected.

Q-Tof MS/MS data were processed using Distiller (Mascot Server 2.4) and interrogated using Mascot MS/MS ion searches [NCBI database, Taxonomy: All entries; Enzyme: Trypsin; Fixed modifications: Pyridylethyl (C); Variable modifications: Gln-> pyro-Glu (N-term Q), Oxidation (M); Peptide mass tolerance ± 0.3 Da; Fragment mass tolerance ± 0.3 Da; Peptide charge 2, 3, and 4+; Instrument Agilent Q-Tof]. Approximate quantification of proteins in PrP precipitates was carried out by spectral counting using the Exponentially Modified Protein Abundance Index (emPAI) (Ishihama et al., 2005) available in Mascot. The RA of a given protein (x) was expressed as its percentage *emPAI* as follows:

$$\%emPAI(x)\;=\frac{emPAI(x)}{\sum_{n=1}^{i}emPAI(i)}\times 100,$$

where *emPAI*(*x*) and *emPAI*(*i*) are the scores provided by Mascot and n the total number of protein families at a set *p*-value; *p* = 0.1 was used here.

LC-mSRM data were acquired and analyte concentrations calculated based on peak area, by interpolation from calibration curves generated, using proprietary software (Agilent MassHunter Quantitative Analysis version B.05.02). All analyte and calibration standard peaks were manually verified and re-integrated as necessary. Linear regression with none or a 1/x weighting was used, whichever gave the better fit for a given peptide (R^2^ > 0.98). Lower limits of detection and quantification (LoDs and LoQs) and imprecision of quantification were established using synthetic peptide mixtures of known concentration (QCs). The LoD was defined as the concentration above which the signal-to-noise ratio exceeded 3. The LoQ was defined for each peptide as the concentration above which the calculated value was within 20% of the theoretical value (*n* = 2 or 3 depending on batch size). LoD and LoQ values for each peptide are given in Table S4.

Calculated peptide concentrations, in duplicate for each sample, were transferred to GraphPad Prism v.6 for the production of N-TAAP plots.

The RA was calculated as a percentage of the summed abundance for 24 possible N-terminal tryptic peptides of PrP^res^ (including pyroglutamyl post-translational modifications), as follows:

$${\text{RA}}_{\text{Npep}}(x)\;=\frac{\left[\text{Npep}(x)\right]}{\sum_{i=1}^{n}\left[\text{Npep}(i)\right]}\;\times 100\%,$$

where Npep(*x*) represents one of the seven N-terminal peptides in the set and Npep(*i*) the concentration of a particular N-terminal peptide.

## Results

### Full-scan analysis of PrP^sc^ and PrP^res^ digests reveals pyroglutamate amino acid residues from the “N-TAAP” region and additional tryptic peptides

#### MS-only analysis

New and previously encountered N-TAAP and core tryptic peptides were revealed in Q-Tof data of three initial TSE tissue preparations (one classical scrapie, one CH1641 scrapie and one ovine BSE) and a recombinant PrP tryptic digest (Figures S1–S3). The data were acquired in MS-only mode and analyzed using Bioconfirm algorithms. A number of the peptide assignments were familiar from previous mSRM analyses and their relative retention times supported the assigned sequences. For the classical scrapie sample without PK treatment, N-terminal cleavage sites H88, G89, G91, and pE95 and tryptic peptides R27-R51, H114-R139, H114-R151, P140-R151, Y152-R159, E154-R159, Y160-K188, pE189-K197, G198-K207, V212-R223, and E224-R231 were suggested. The N-TAAP peptide G85-K109 was additionally found for the PK treated sample. Analogous results were obtained for the preparations from CH1641 scrapie and ovine BSE, which additionally revealed I208-R211. N-terminal tryptic peptides K26-R40 and Y41-R51 were abundant in a digest of recombinant PrP, but not in PrP^Sc^ purified from tissue, even when PK treatment was omitted.

**Figure 2:**
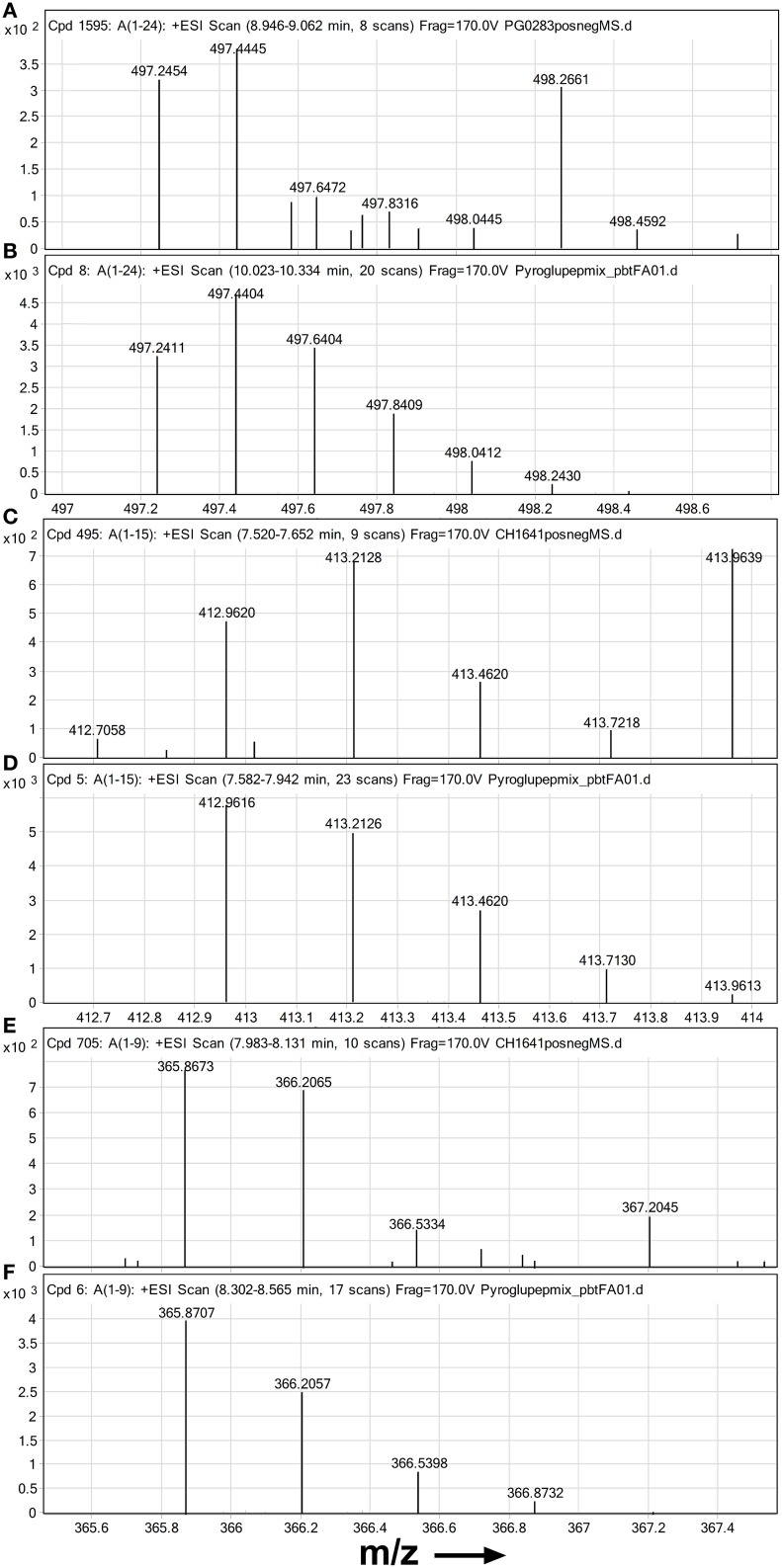
**High resolution mass spectra of ovine PrP peptides with pyroglutamate N-termini**. Mass spectra are shown zoomed in on the isotope distribution of **(A,B)** quintuply protonated pEPHGGGWGQGGSHSQWNKPSKPK (pE86-K109) **(A)** suspected in PrP^Sc^ digest from classical scrapie infected brain sample and **(B)** synthetic standard; **(C,D)** quadruply protonated pEGGSHSQWNKPSKPK (pE95-K109) and **(E,F)** triply protonated pEWNKPSKPK **(C,E)** suspected in PrP^Sc^ digest from an experimental CH1641 scrapie brain sample and **(D,F)** synthetic standards. Chip-HPLC Q-Tof mass spectra were obtained by integrating the total MS signal over the full width at base of the chromatographic peak of the peptide. Spectra **(B,D,F)** were based on 100 fmol on column of each standard.

Most notable amongst novel PrP fragment suggestions were the pyroglutamyl post-translational modifications of peptides with N-terminal glutamines. The presence of these pyroglutamyl compounds was supported by the isotope patterns of their most abundant charge states in mass spectra summed over the time interval of their chromatographic peaks (Figures 2A,C,E). A comparison to corresponding synthetic standards (Figures 2B,D,F) gave similar retention times and isotope distributions. Peptide pE95-K109 was identified at a retention time of 7.5 min in CH1641 scrapie without PK treatment (Figure 2C and Figure S2A). The BioConfirm assignment of pE95-K109 to a peak at a retention time of 10.6 min in the data from the classical scrapie sample, appeared to be incorrect: apart from the large discrepancy in retention time, the pE95-K109 standard was found predominantly in the 4+ charge state (Figure 2D), while the compound at 10.6 min was present in 2+ and 3+ charge states. The BioConfirm assignment of pE101-K109 to a compound at 8.2 min in the CH1641 (Figure 2E and Figure S2A) and BSE samples (Figure S3C) was supported based on its synthetic peptide (Figure 2F). Although not revealed following Bioconfirm processing and analysis, signal corresponding to peptide pE86-K109 was found in the full scan mass spectra of the classical scrapie digest without PK treatment (Figure 2A) with similar isotope pattern to the synthetic version (Figure 2B).

Pyroglutamyl modified tryptic peptides pE189-K197 (samples and recPrP, Figures S1–S4), pE189-K207 and pE189-R211 (recPrP, Figure S4) were also detected. Given that these peptides were unlikely to be present prior to digestion with trypsin, these were most likely to be acid-induced artifacts. While application of the Bioconfirm algorithm facilitated the detection of many PrP-derived peptides, identification of Q189-K197 was less straightforward, possibly due to interfering signal; hence this compound is not displayed in the Bioconfirm-based extracted compound chromatograms (Figures S1–S4). When we applied extracted ion chromatogram searches for m/z 508.7727 and m/z 339.5177, corresponding to the doubly and triply protonated monoisotopic ions of Q189-K197, we did manage to retrieve peaks at the expected retention time.

#### MS/MS analysis

In first instance, we seemed unable to identify ovine prion protein in brain tissue preparations in individual data files obtained by Q-Tof auto-MS/MS analysis. However, a multi-file Mascot database search (*p* = 0.1) of all samples resulted in identification of ovine prion protein in 26th place out of 36, with a Protein Family Summary score of 34 based on matches of G198-K207, V212-R223, and E224-R231 (+PK samples) resp 52nd place out of 74, scoring 40 based on matches of V212-R223 and E224-R231 (−PK samples). This corresponded to a RA (%emPAI) of 0.89% and 0.38%, respectively. The highest scoring protein families were Ig lambda light chain (19%) and ferritin light chain (14% in the +PK samples and beta-globin (22%) and ferritin heavy chain (9%) in the −PK samples. A similar search of MS/MS analysis of recombinant PrP resulted ovine PrP ranking top with a score of 244, based on matches of eight PrP peptides, resulting in a sequence coverage of 44% of the final protein product. Here its %emPAI was 63%, that of trypsin 32% and keratin 4%. No prion protein could be identified in the control tissue preparation; the most abundant protein families were beta tubulin and beta globin (−PK) and ferritin heavy and light chain (+PK).

### *In vitro* N-terminal pyroglutamyl modification can be minimized by rapid analysis

**Figure 3:**
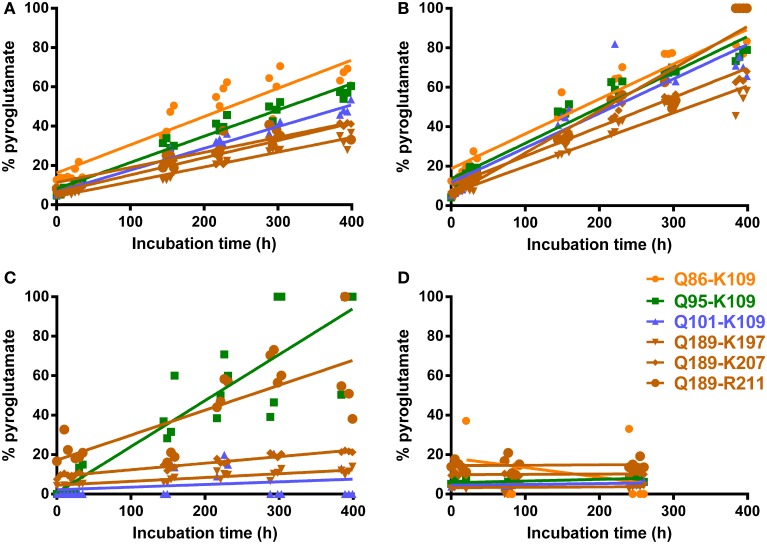
**Conversion rates of N-terminal glutamine to pyroglutamate for six PrP peptides**. Chip-HPLC Q-Tof mass spectrometry was used. Repeat analyses of N-terminal glutamine peptide mixtures in water, PTB-FA and PBT-HCl, spaced by buffer blanks, were run and the injection times recorded as described in Materials and Methods. **(A)** Post-trypsin buffer (PTB) to which 0.5 volume 5% formic acid was added as by PrP^Sc^ digest preparation protocol (final pH 3.0) **(B)** PTB plus 0.5 volume 5% HCl (pH 1.0) **(C)** PTB + 0.5 volume water (pH 8.0) **(D)** peptides dissolved in pure water (pH 7.0). Q-pE conversion rates calculated from these plots are given in Table 1.

To assess whether the identification of pyroglutamyl PrP peptides in full-scan mass spectrometric analysis of PrP^res^ could be a sample preparation artifact introduced by acidification alone, we determined the rate of conversion for a number of PrP peptides with N-terminal glutamine under acidic, non-buffered and more basic (pH = 8) conditions, at a temperature of 8°C as routinely maintained in the autosampler compartment. Addition of FA at the end of tryptic digestion results in a post-trypsin buffer solution of pH = 3 (PTB-FA). Reasoning that perhaps a weak acid such as FA but not a strong acid such as HCl might catalyze the *in vitro* conversion of N-terminal glutamine to pyroglutamate (Chelius et al., 2006), we wondered if adding 5% hydrochloric acid, which would result in a solution of pH = 1 (PTB-HCl), might reduce or prevent the *in vitro* conversion of glutamine to pyroglutamate. In addition we determined conversion rates in HPLC grade water (pH = 7) and tryptic digestion buffer to which 0.5 volume water was added instead of acid (PTB-water). Repeated analysis of synthetic peptide preparations in each of these solutions allowed the corresponding conversion rates to be determined (Figure 3 and Table 1).

**Table 1:** **N-terminal glutamine to pyroglutamate conversion rates (%/h) for PrP peptides (see Figure 1 for sequences) in various media at 8°C**.

		**Q86-K109**	**Q95-K109**	**Q101-K109**	**Q189-K197**	**Q189-K207**	**Q189-R211**
PTB-FA	Slope	0.14 ± 0.01	0.13 ± 0.01	0.11 ± 0.003	0.08 ± 0.002	0.09 ± 0.003	0.08 ± 0.01
	*R*^2^	0.8745	0.9692	0.9778	0.9673	0.9689	0.7083
PTB-HCl	Slope	0.18 ± 0.01	0.18 ± 0.01	0.17 ± 0.01	0.14 ± 0.01	0.15 ± 0.005	0.21 ± 0.02
	*R*^2^	0.9307	0.9428	0.8782	0.9529	0.9776	0.9109
PTB-water	Slope	−0.05 ± 0.06	0.009 ± 0.004	0.004 ± 0.001	0.002 ± 0.003	0.001 ± 0.002	0.002 ± 0.008
	*R*^2^	0.1004	0.2997	0.3031	0.02758	0.03721	0.005692
Water	Slope	No data	0.23 ± 0.02	0.01 ± 0.01	0.02 ± 0.002	0.03 ± 0.002	0.13 ± 0.02
	*R*^2^		0.8041	0.06837	0.7628	0.9538	0.5798

*PTB, post-trypsin buffer*.

While the abundance of peptides with N-terminal pE was negligible immediately following addition of acid to buffers containing N-terminal glutamyl peptides, this changed over time. We found that in 400 h approximately 40% of glutamine was converted to pE for peptide solutions to which FA had been added, which corresponds to 3% over a 24-h period. For solutions to which HCl was added, this conversion rate was increased to 4.3% in 24 h. Under non-buffered or basic conditions, conversion rates remained under 0.1%. The MS response of peptides dissolved in HPLC-grade water alone was much lower than in any of the other solvents to the extent that no data could be obtained for the longest N-TAAP peptide (Q86-K109). Even though the HPLC loading, washing and elution solvents all contained 0.1% FA, ion formation may have been considerably poorer for water-dissolved standards compared to acidified and non-acidified post-trypsin buffer or lack of buffer components in the sample solvent may have made the peptides more prone to strong adsorption within the HPLC system. Similar analysis of mixtures of pyroglutamyl peptide standards did not show changes such as reversion to glutamine in any of the same four solvents.

The low conversion rates at pH = 8.0 suggests that *in vitro* pE formation is unlikely to commence prior to the addition of acid in the final step of the digest preparation. Thus, N-terminal pyroglutamyl artifacts can be minimized by keeping the amount of time between addition of acid and analysis of a preparation to a minimum. To allow automated overnight HPLC MS, a limit of 18 h was set for the time preparations were allowed to spend in the autosampler compartment following their acidification. This corresponds to a 2.5% conversion of glutamyl into pyroglutamyl peptides in PTB-FA. Acidified preparations that could not be analyzed within 18 h were stored at −20°C immediately following acidification and placed in the autosampler compartment shortly before analysis. Similarly, batches of calibration and QC standards were aliquoted and stored at −20°C until required in an assay.

### The relative abundance of pyroglutamyl N-TAAP peptides in ovine TSEs is different between TSEs and animals of different geographical origins

**Figure 4:**
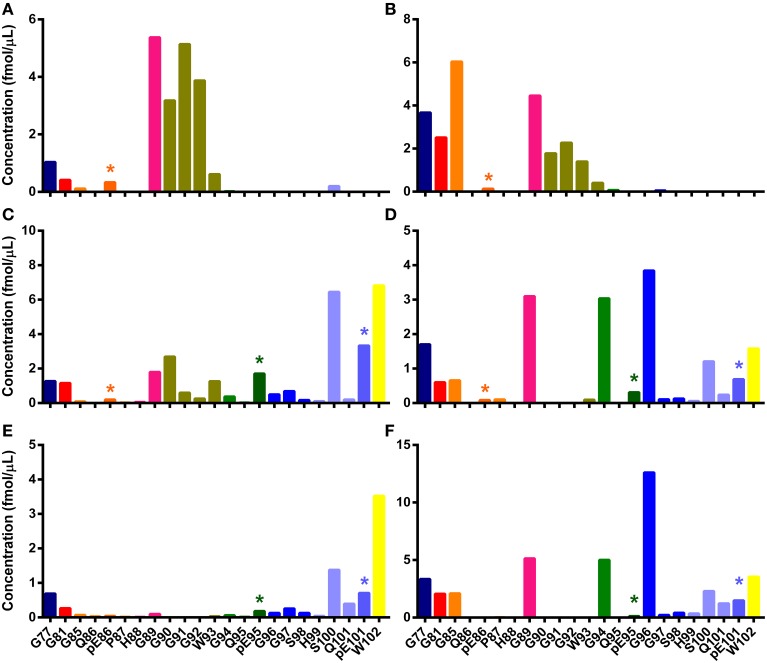
**N-TAAPs from ovine TSEs**. Graphs show calculated concentrations (±SD) of N-TAAP peptides determined by chip-HPLC SRM mass spectrometry of digest preparations from the brain stem of individual animals. **(A,B)** Classical scrapie: VRQ/VRQ Swaledale, neutered male, homebred; **(C,D)** Experimental CH1641 scrapie: AHQ/AHQ Cheviot, neutered male; **(E,F)** Experimental BSE: ARQ/ARQ Romney. **(A,C,E)** without proteinase K treatment; **(B,D,F)** with proteinase K treatment. Asterisks are used to point out the peptides with pyroglutamyl N-termini.

Once the mSRM prion peptide assay was developed and acidification protocols established, they were used to analyze sample digests. The mSRM chromatograms (Figures S5–S7) of the same PrP^Sc^ digests used in the analyses described above were found to be comparable to the Q-Tof-based extracted compound chromatograms (Figures S1–S3). The concentrations of the PrP peptides were determined and N-TAAPs (Figure 4) and tryptic peptide profiles (Figure S8) established. Pyroglutamyl N-TAAP peptides were identified in these samples, albeit at relatively low abundance. Detection of specific N-terminal cleavages in the preparations without PK treatment was confirmed: G89, G90, G91, G92 were the four most abundant N-terminal cleavage sites for classical scrapie (Figure 4A) and S100, pE101, and W102 the three most abundant ones for CH1641 scrapie (Figure 4C) and ovine BSE (Figure 4E).

**Figure 5:**
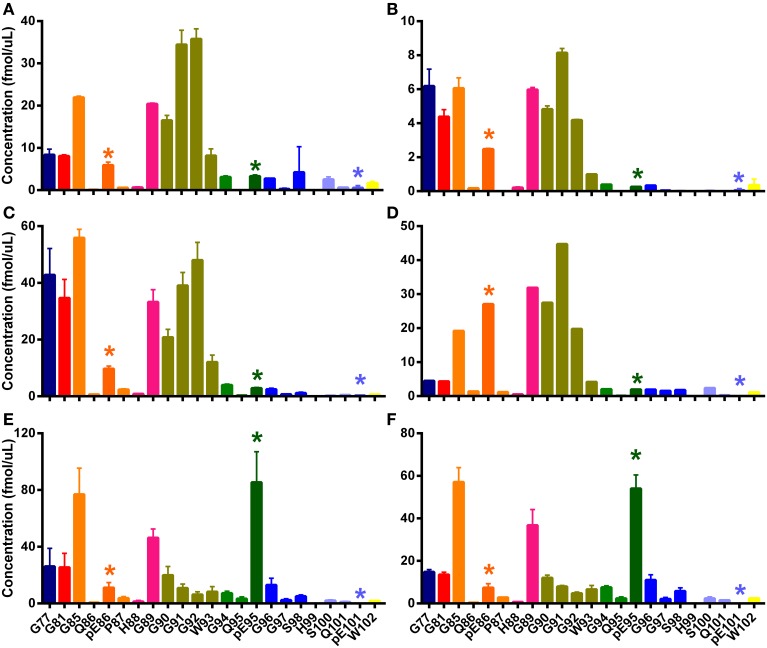
**N-TAAPs from natural classical scrapie samples**. Graphs show calculated concentrations (±SD) of N-TAAP peptides determined by chip-HPLC SRM mass spectrometry of digest preparations from the brain stem of individual animals, obtained by processing 2 × 1.75 ml of 10% homogenate with PK treatment. **(A)** ARQ/ARQ Swaledale, neutered male, homebred; **(B)** VRQ/VRQ Swaledale, female, homebred; **(C)** ARH/VRQ Texel, female, purchased into flock from farm A; **(D)** ARQ/VRQ white-faced Dartmoor, female, homebred **(E,F)** ARQ/ARQ Vendeen, female, purchased into flock from farm B. Asterisks are used to point out the peptides with pyroglutamyl N-termini.

**Figure 6:**
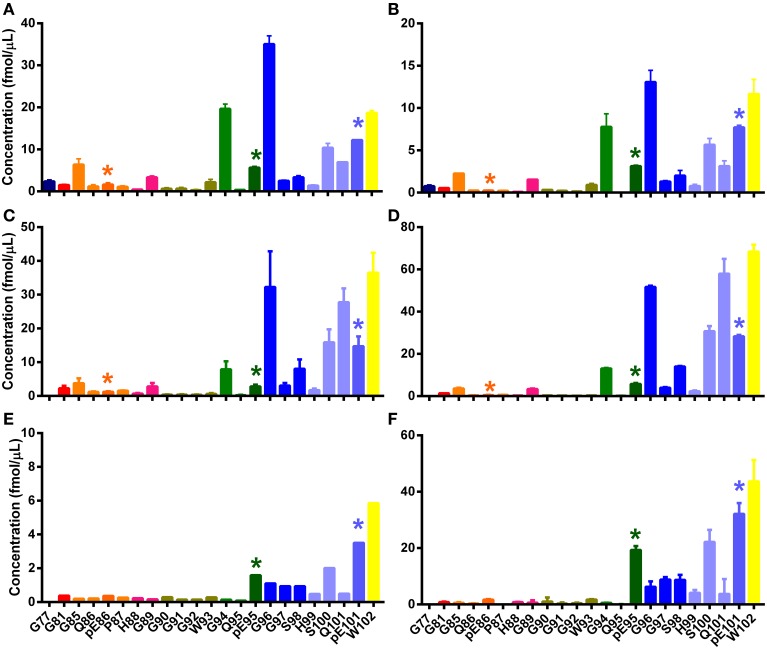
**N-TAAPs from experimental ovine TSEs**. Graphs show calculated concentrations (±SD) of N-TAAP peptides determined by chip-HPLC SRM mass spectrometry of digest preparations from the brain stem of individual animals, obtained by processing 2 × 1.75 ml of 10% homogenate. All animals were neutered males of the Cheviot breed, AHQ/AHQ genotype. **(A,B)** PK treated CH1641 scrapie (Coombelands farm, Surrey); **(C,D)** PK treated ovine BSE (ADAS Drayton, Warwicksire); **(E,F)** ovine BSE samples corresponding to **(C,D)** without PK treatment. Asterisks are used to point out the peptides with pyroglutamyl N-termini.

Peptide abundances were thus determined in sample digests from a further six classical scrapie infected animals of various breeds, genotypes and origin kept in the APHA experimental flock (Ryder et al., 2004). The resulting N-TAAPs showed that, while the most abundant PK cleavage sites were generally in the region between G77 and G94, the RA of these sites could vary considerably between the samples (Figure 5). Three of the samples, from a homebred, ARQ/ARQ Swaledale neutered male (Figure 5A), a homebred VRQ/VRQ Swaledale female (Figure 5B), and a purchased ARH/VRQ Texel female (Figure 5C), contained similar amounts of regular N-TAAP peptides and small amounts of pyroglutamate N-terminal residues pE86 and pE95, with pE86 being somewhat more abundant. In these cases, the abundance of G90, G91, G92, and W93 was similarly high. The profile for a homebred ARQ/VRQ white-faced Dartmoor female (Figure 5D) was also similar, except for its pE86 abundance which was notably higher. In contrast, the N-TAAP profiles from two purchased ARQ/ARQ Vendeen females (Figures 5E,F) contained relatively low amounts of pE86, G90, G91, G92, and W93 N-termini, while the abundance of pE95 was remarkably high. Additional preparations of experimental CH1641 scrapie and ovine BSE, from two animals each of the same breed (Cheviot) and genotype (AHQ/AHQ), were also analyzed. Cleavage sites pE95 (but not Q95), S98, Q101 and pE101 were detected abundantly (Figures 6A–D). These sites were found in addition to previously identified cleavage sites G94, G96, S100, and W102 for these TSEs (Tang et al., 2012). The N-TAAP profiles show that the distribution of pE cleavage sites for CH1641 and BSE is further toward the C-terminus compared to classical scrapie, consistent with the general trend that the ragged N-terminus is further C-terminal for these TSEs. Q101 was robustly detected for both CH1641 scrapie and ovine BSE. Although qualitatively similar, the abundances of most of these cleavage sites were significantly different between CH1641 scrapie and BSE: unpaired *t*-tests carried out following calculation of the relative abundance (RA, Methods 2.4) of each of the N-TAAP peptides compared between the TSEs, resulted in the following *p*-values: G94: *p* < 0.0001; G96: *p* = 0.0340; S98: *p* = 0.048; S100: *p* = 0.0112; Q101:*p* < 0.0001; pE101: *p* = 0.3387; W102: *p* = 0.0039.

Digests of the same BSE samples without PK treatment (Figures 5E,F) showed pE95, S100, pE101, and W102 N-terminal fragments, while cleavage sites G96 and Q101 were much less abundant, confirming the results shown in Figure 4. Signal from the other samples shown in Figures 5, 6 was insufficient to give rise to N-TAAP profiles without PK addition; however, low-level signal from pE95 N-TAAP peptides could be detected in the samples where pE95 was also abundant following PK treatment (Figures 4E,F).

### Tryptic peptide profiles allow determination of allele frequencies and show R-P bond cleavage

#### Allele frequencies

The relative involvement of the protein products of PrP alleles implicated in the susceptibility and survival time of sheep exposed to prions has been speculated for decades but the lack of allotype-specific mAbs or other methods of discrimination at the protein level of allelic variants has hindered their investigation. Our MS methodology now allows this to be resolved. Tryptic peptides containing both A136 and V136 allotypes were observed in the tryptic peptide profiles from PrP heterozygous animals, while only V136 or A136 peptides were observed in samples from homozygous animals (Figures S8, S9). In the samples from heterozygous animals (S9C and S9D), the ratios of the corresponding peptides differed from unity: for the ARQ/VRQ sample (Figure S9D), V136 peptides were overall 7.5 times more abundant than A136 peptides, while for the VRQ/ARH, V136 peptides were 5.8 times more abundant than A136 peptides. Interestingly, from the same VRQ/ARH sample peptides containing Q171 were 3.0 times more abundant than those containing H171.

#### R-P bond cleavage

The cleavage of the arginyl-proline bond by trypsin in peptides and proteins is considered rare but, in most of our prion protein samples, the peptides H114-R139 and P140-R151 were detected in good abundance, indicating that the R139-P140 bond is cleaved with reasonable frequency. The RA of fragments cleaved and not cleaved between R139-P140 was found to vary considerably between samples and would presumably depend on the total amount of protein present. By contrast, cleavage between R167-P168 was observed much less frequently: only very small amounts of the peptide Y160-R167 could be detected, mostly in samples with a high PrP^Sc^ content.

## Discussion

Pyroglutamyl-modified QHTVTTTTK, which corresponds to the peptide Q189-K197 in sheep PrP, had been identified previously by mass spectrometry following denaturation and endoproteinase Lys-C digestion of the protease-resistant core of hamster PrP^Sc^ and was considered an *in vitro* acid-induced modification without pathological significance (Baldwin et al., 1990). We had ourselves observed a low-abundance pE189 peptides in addition to Q189 peptides in freshly prepared tryptic digests of ovine PrP^res^ (Figures S8, S9) and found that the abundance of pE189 peptides increased when samples were repeatedly analyzed and, as described by others previously (Baldwin et al., 1990; Stahl et al., 1993), we considered the Q189/pE189 ratio a useful parameter to assess the level of in conversion of Q into pE. During the development of our N-TAAP analysis methodology and its use as an epidemiological tool to identify TSE strains and subtypes from naturally infected scrapie cases (Gielbert et al., 2013), we observed that the ratio of total N-TAAP peptides to core- and C-terminal tryptic peptide concentrations was considerably different from unity. Our subsequent investigation to expand the N-TAAP system to map all possible N-termini of PrP^Sc^, and improve its resolution as an indicator of tertiary and quartenary structure led us to discover a much larger sub-set of pyroglutamyl-N-terminal PrP peptides in scrapie-infected sheep brain and prompted the question: natural or artifact? This led us to investigate more closely the rate and conditions of N-terminal glutamine cyclisation to a pyrrolidine carboxylate (pyroglutamyl) residue.

### *In vitro* studies

Using synthetic peptides, we found the *in vitro* conversion of N-terminal glutamine to pyroglutamate residues, at a given buffer composition, pH and temperature, to occur at a constant rate independent of the peptide sequence or its amino-acid composition. This process may occur in the time lag following digest preparation and analysis by HPLC-mSRM and when not kept to a minimum could give rise to inconsistent results and artifacts. We found that the conversion rates were sufficiently low to give a window of analysis of several hours between acidification and injection on column of tissue extracts and this allowed us to develop a work process allowing the RAs of pE–peptides to remain representative of the initial sample.

The conversion rates were found to be higher in buffer with added HCl than in buffer with added FA; therefore the conversion rate more likely correlates with the overall pH rather than depends on the nature of the acid added. This differs from what has been described in the literature (Chelius et al., 2006) but may be a consequence of the fact that we used a buffer rather than HCl alone. Use of ultrapure water alone as a solvent for the PrP peptides gave very limited signal in the HPLC-MS analysis and we found that buffering the analyte solution is important to facilitate peptide detection by electrospray mass spectrometry even if this gives a pH higher than neutral.

### Analysis of pE-PrP peptides from sheep

The introduction of pE-PrP peptides into the N-TAAP analysis allowed us to observe remarkable differences in the relative amounts of pE-PrP in sheep with different types of TSEs. While classical scrapie samples contained in a majority pE86- and pE95-PrP fragments, pE95 and pE101 fragments were the most abundant in CH1641 scrapie and ovine BSE samples. This is consistent with previous work showing the PrP^res^ from PK-treated CH1641 scrapie and BSE digests is smaller in size to the PrP^res^ of classical scrapie cases and more truncated at the N-terminus. Moreover, different ratios of pE86 and pE95 N-terminal fragments were present in scrapie samples from animals of different breed and geographical origin which suggests that relative quantitation of these peptides may be used as a more generic marker for TSE typing.

No Q86-K109 and Q95-K109 N-TAAP peptides were found above their detection limits for any of the TSE cases examined but Q101-K109 could be detected in both ovine BSE and CH1641 scrapie PrP^res^ and provided a direct confirmation from our work of the reported greater N-terminal sensitivity of the PrP^Sc^ of these types of TSE compared to that from classical scrapie.

The variable but ubiquitous nature of these pE peptides support the idea that they might be directly involved in the prion-like activity of PrP^Sc^ (by, for example, promoting or changing the rate of protein aggregation) and that the RA of various pyroglutamyl N-terminal cleavage sites could also be valuable parameters in TSE discrimination. Variable rates of PrP misfolding, aggregation and degradation are the likely basis for phenotypic (or strain) differences in prion-affected animals and our Q-Tof MS approach allows the simultaneous investigation of factors such as post-translational modification (pE formation), conformation (by N-TAAP analysis) and amino-acid polymorphisms (allotype ratio, see below) which affect the kinetics of these proteostatic processes.

### General identification of PrP peptides by Q-ToF MS and MS/MS analysis

The PrP peptides we identified most frequently using auto-MS/MS and database searching were Q189-K197, V212-R223, and E224-R231, implying that these peptides are the most proteotypic–the most likely to be identified by a particular MS platform in quantitative proteomics of complex mixtures (Mallick et al., 2007). Only MS/MS analysis of recombinant PrP resulted in identification of additional peptides such as E155-R159 and H114-R151. These and other PrP fragments could be identified based on accurate mass only, by applying the proprietary deconvolution and search algorithms built in to Agilent Qualitative analysis Bioconfirm software. The MS-only approach allowed identification of additional peptides even if their fragmentation pattern was not amenable to identification by database search. Using this process, we were able to assign nearly complete sequence coverage for the recombinant PrP outside of the very long peptide containing the N-TAAP region (Y52-K109).

MS/MS analysis of PrP^Sc^ and PrP^res^ tryptic digests with emPAI-based quantification indicated that the abnormal prion protein was a relatively small fraction in our preparations. This contrasts with findings by others, who if at all, report co-purified proteins at much lower levels (Baldwin, 2001; Morel et al., 2007; Howells et al., 2008) using mass spectrometry, where PrP^res^ appears to be a major component of the final preparation. Edman N-terminal sequencing of hamster, mouse and cattle PrP^Sc^ and PrP^res^ also identified the major component as abnormal prion protein purified by differential centrifugation, gel filtration and gel electrophoresis elution (Hope et al., 1986, 1988a,b) to be the prion protein. These latter studies would not have detected the pyroglutamyl-PrP in cattle, mouse or hamster brain, as the Edman sequencing process is blocked by pyroglutamate N-termini. Mass spectrometry-based analysis however is capable to detect pyroglutamylated peptides.

The emPAI-based determination of PrP^Sc^/PrP^res^ abundance in the preparations may be biased because the number of proteotypic peptides of PrP may be below average and this may have been exacerbated by the high tissue/solvent ratios we have developed in our N-TAAP methodology compared to earlier studies. Nonetheless, good quantities of PrP peptides were generally detected in our preparations particularly using mSRM methods. These were developed using peptide standards to determine retention time, charge state and fragmentation pattern, even if its fragmentation pattern makes it not particularly amenable to identification by database searching. In our experience, very short (<5 residues) or very long (>20 residues) tryptic peptides provide insufficient proteotypic information while the N-TAAP peptides with N-terminal glutamine residues predominantly lose water in addition to a minor fragment. Nonetheless, based on the combination of fragmentation and retention time features, all these peptides were detectable using mSRM. This allowed identification of nearly all PrP peptides even from tissue preparations, underlining the power of mSRM to allow determination and quantification of peptides from complex mixtures.

The Q-Tof MS-only extracted compound chromatograms (Figures S1–S4) and QQQ mSRM chromatograms (Figures S6–S8) acquired from the same samples show most of the PrP peptides at comparable elution times. Nonetheless, not all PrP peptides detectable by mSRM could be extracted from the Q-Tof MS-only data, particularly multiply protonated peptides such as G77-K109 and G81-K109 (6 and 5+) and H114-R154 (6+), even though up to 15 charges were allowed in the “find by molecular features” algorithm. The combination of high charge state, low abundance, mass inaccuracies and noise may preclude these ions from being recognized by the software.

LoDs and LloQs were found to vary for the different peptides, their individual properties influencing their interaction with the HPLC column, ionization response and fragmentation properties. LloQ for Q189-K197 was relatively high presumably because its retention was not as reliable as that for more hydrophobic peptides, particularly when present at high concentration. Peptides G77-K109 and G81-K109 are prone to tailing on elution from high performance column chromatography columns as they are relatively long, with 33 and 29 residues respectively, and contain 6 and 5 basic amino acid residues. Peptides H114-R139, -R151, and -R154 are relatively hydrophobic and prone to carry over between runs, again increasing limits of detection and quantification.

### Analysis of PrP^sc^ and PrP^res^, and their tryptic peptides

While the highest abundance of N-TAAP fragments was found in PK-treated samples (PrP^res^), a notable number of N-terminal fragments were identified in digests prepared without PK treatment (PrP^Sc^). The N-TAAP patterns of PrP^Sc^ and PrP^res^ differ even when prepared from the same TSE case. For the classical scrapie sample (Figures 4A,B), PK treatment results in increased abundance of G85, which corresponds to a cleavage further N-terminal than the fragments present without PK treatment. Similarly, for ovine BSE (Figures 6C–F), the PK treated digests contain a higher proportion of G94, G96, and Q101, which are on balance N-terminal to pE95, S100, pE101, and W102 present in the -PK preparation. Safar and colleagues have shown that variable proportions of abnormal prion protein in TSE-affected brain may be proteinase-sensitive and these proportions vary in brain infected by different strains of TSE (Kim et al., 2012); similar strain-specific variability in the detergent-solubility of PrP^Sc^ has also been described (Hope and Kirby, 2012) and our detergent-based extraction of PrP^Sc^ and its analysis by N-TAAP has the potential to type TSEs using these under-investigated characteristics of the prion.

Q-Tof MS data of recombinant PrP gave good signals for K26-R40 and Y41-R51 but little or none of these N-terminal tryptic peptides were obtained from digests of PrP^Sc^ semi-purified from brain tissue. Analysis by mSRM was unable to identify much Y41-R51 from any sample, and the yield was certainly much lower than the amount of tryptic peptides obtained from the protease resistant core. We interpret this as evidence for the continued action of endogenous tissue proteases in the tryptic digestion of the semi-purified brain material which identifies this stage of the process as a key point for further method development.

Tryptic peptides H114-R139 and H114-R151 could be detected both in PrP^Sc^/PrP^res^ isolated from TSE infected tissue (Figures S1–S3) and in recombinant PrP (Figure S4). Tryptic cleavage rates after arginine or lysine residues followed by proline are generally much lower than when followed by any other amino acid residue; however the R139-P140 site appears to be an exception to this rule. The RA of H114-R139 was found to be lower in the recombinant PrP preparation. The nearly complete absence of Y160-R167 (and P168-K188) compared to Y160-K188, in both recombinant PrP and preparations from tissue, indicates that the R167-P168 site is cleaved less frequently than R139-P140.

Quantification of core tryptic PrP^Sc^ and PrP^res^ peptides allowed the determination of allotype ratios in abnormal prion protein from PrP heterozygous sheep. In principle, the tryptic peptide profiling assay can determine A/V136, L/F41, H/R154, and H/Q/R171 ratios. In the present work, three A/V136 ratios and one H/Q171 ratio were determined for classical scrapie. Our A/V ratio, was 1/7.5 for ARQ/VRQ genotype samples, and qualitatively similar, if quantitatively different, the 1/3 ratio determined using H^18^_2_O trypsin digestion and Maldi-Tof analysis calibrated by recombinant protein digests (Morel et al., 2007). The quantitative differences may be due to the particular types of classical scrapie examined, biological variation, or due to the biochemistry: the frequency of trypsin cleavage at the R139-P140 site does not reproduce easily, particularly between recombinant PrP protein standards and PrP^Sc^ purified from samples. Interestingly, the A/V136 and H/Q171 ratios that we determined here for a VRQ/ARH genotype sample differed by a factor two. While these ratios might be expected to be equal, they may differ because the tryptic peptide that includes H171 also includes glycosylation site N184, and variable N-glycosylation of N184 is expected to affect the yields of the H171 containing peptide.

In summary, in this work we have determined the presence of N-terminally pyroglutamylated PrP^Sc^ fragments in preparations of ovine scrapie-infected brain and demonstrated that the rate of *in vitro* conversion of N-terminal glutamine to pyroglutamate cannot on its own account for these. Pyroglutamylated peptides were identified both under +PK and −PK conditions, and therefore must have existed *in vivo*. Furthermore, not only have differences in RA of various N-terminally truncated pyroglutamylated residues been observed between known TSE strains, but also between scrapie-infected animals of different origins and possibly scrapie sub-types. To the best of our knowledge, this is the first time that pyroglutamylated N-terminally truncated PrP^Sc^ peptide fragments formed *in vivo* have been demonstrated in TSE samples. While our data indicate that the RA of various pyroglutamyl N-terminal cleavage sites could be valuable parameters in TSE discrimination, it would also be worthwhile, given that pyroglutamylated fragments of amyloidogenic proteins have been implicated in causing or accelerating protein aggregate formation and aggravating disease symptoms (Momoi et al., 1995; Schilling et al., 2006, 2008; Schlenzig et al., 2009; Wirths et al., 2009; Jawhar et al., 2011b; Nussbaum et al., 2012; Saul et al., 2013; Matos et al., 2014), to investigate whether pE PrP fragments may play a role in promoting or changing the rate of PrP^Sc^ aggregation.

### Conflict of interest statement

The authors declare that the research was conducted in the absence of any commercial or financial relationships that could be construed as a potential conflict of interest.
