# Supplementary material for: Pyroglutamyl-N-terminal prion protein fragments in sheep brain following the development of transmissible spongiform encephalopathies
Source: Front Mol Biosci. 2015 Mar 11;2:7. doi: 10.3389/fmolb.2015.00007 (PMC4429639; doi:10.3389/fmolb.2015.00007)

Table S1: semi-tryptic (potential N-TAAP) ovine PrP peptides used to interrogate full-scan Q-Tof analysis of PrP^{res} preparations using MassHunter Qualitative with BioConfirm

	peptide abbreviation	sequence
1.	Y52-K109	YPPQGGGGWGQPHGGGWGQPHGGGWGQPHGGGWGQPHGGGGWGQGGSHSQWNKPSKPK
2.	P53-K109	PPQGGGGWGQPHGGGWGQPHGGGWGQPHGGGWGQPHGGGGWGQGGSHSQWNKPSKPK
3.	P54-K109	PQGGGGWGQPHGGGWGQPHGGGWGQPHGGGWGQPHGGGGWGQGGSHSQWNKPSKPK
4.	Q55-K109	QGGGGWGQPHGGGWGQPHGGGWGQPHGGGWGQPHGGGGWGQGGSHSQWNKPSKPK
5.	G56-K109	GGGGWGQPHGGGWGQPHGGGWGQPHGGGWGQPHGGGGWGQGGSHSQWNKPSKPK
6.	G57-K109	GGGWGQPHGGGWGQPHGGGWGQPHGGGWGQPHGGGGWGQGGSHSQWNKPSKPK
7.	G58-K109	GGWGQPHGGGWGQPHGGGWGQPHGGGWGQPHGGGGWGQGGSHSQWNKPSKPK
8.	G59-K109	GWGQPHGGGWGQPHGGGWGQPHGGGWGQPHGGGGWGQGGSHSQWNKPSKPK
9.	W60-K109	WGQPHGGGWGQPHGGGWGQPHGGGWGQPHGGGGWGQGGSHSQWNKPSKPK
10.	G61-K109	GQPHGGGWGQPHGGGWGQPHGGGWGQPHGGGGWGQGGSHSQWNKPSKPK
11.	Q62-K109	QPHGGGWGQPHGGGWGQPHGGGWGQPHGGGGWGQGGSHSQWNKPSKPK
12.	P63-K109	PHGGGWGQPHGGGWGQPHGGGWGQPHGGGGWGQGGSHSQWNKPSKPK
13.	H64-K109	HGGGWGQPHGGGWGQPHGGGWGQPHGGGGWGQGGSHSQWNKPSKPK
14.	G65-K109	GGGWGQPHGGGWGQPHGGGWGQPHGGGGWGQGGSHSQWNKPSKPK
15.	G66-K109	GGWGQPHGGGWGQPHGGGWGQPHGGGGWGQGGSHSQWNKPSKPK
16.	G67-K109	GWGQPHGGGWGQPHGGGWGQPHGGGGWGQGGSHSQWNKPSKPK
17.	W68-K109	WGQPHGGGWGQPHGGGWGQPHGGGGWGQGGSHSQWNKPSKPK
18.	G69-K109	GQPHGGGWGQPHGGGWGQPHGGGGWGQGGSHSQWNKPSKPK
19.	Q70-K109	QPHGGGWGQPHGGGWGQPHGGGGWGQGGSHSQWNKPSKPK
20.	P71-K109	PHGGGWGQPHGGGWGQPHGGGGWGQGGSHSQWNKPSKPK
21.	H72-K109	HGGGWGQPHGGGWGQPHGGGGWGQGGSHSQWNKPSKPK
22.	G73-K109	GGGWGQPHGGGWGQPHGGGGWGQGGSHSQWNKPSKPK
23.	G74-K109	GGWGQPHGGGWGQPHGGGGWGQGGSHSQWNKPSKPK
24.	G75-K109	GWGQPHGGGWGQPHGGGGWGQGGSHSQWNKPSKPK
25.	W76-K109	WGQPHGGGWGQPHGGGGWGQGGSHSQWNKPSKPK
26.	G77-K109	GQPHGGGWGQPHGGGGWGQGGSHSQWNKPSKPK
27.	Q78-K109	QPHGGGWGQPHGGGGWGQGGSHSQWNKPSKPK
28.	P79-K109	PHGGGWGQPHGGGGWGQGGSHSQWNKPSKPK
29.	H80-K109	HGGGWGQPHGGGGWGQGGSHSQWNKPSKPK
30.	G81-K109	GGGWGQPHGGGGWGQGGSHSQWNKPSKPK
31.	G82-K109	GGWGQPHGGGGWGQGGSHSQWNKPSKPK
32.	G83-K109	GWGQPHGGGGWGQGGSHSQWNKPSKPK
33.	W84-K109	WGQPHGGGGWGQGGSHSQWNKPSKPK
34.	G85-K109	GQPHGGGGWGQGGSHSQWNKPSKPK
35.	Q86-K109	QPHGGGGWGQGGSHSQWNKPSKPK
36.	P87-K109	PHGGGGWGQGGSHSQWNKPSKPK
37.	H88-K109	HGGGGWGQGGSHSQWNKPSKPK
38.	G89-K109	GGGGWGQGGSHSQWNKPSKPK
39.	G90-K109	GGGWGQGGSHSQWNKPSKPK
40.	G91-K109	GGWGQGGSHSQWNKPSKPK
41.	G92-K109	GWGQGGSHSQWNKPSKPK
42.	W93-K109	WGQGGSHSQWNKPSKPK
43.	G94-K109	GQGGSHSQWNKPSKPK
44.	Q95-K109	QGGSHSQWNKPSKPK
45.	G96-K109	GGSHSQWNKPSKPK
46.	G97-K109	GSHSQWNKPSKPK
47.	S98-K109	SHSQWNKPSKPK
48.	H99-K109	HSQWNKPSKPK
49.	S100-K109	SQWNKPSKPK
50.	Q101-K109	QWNKPSKPK
51.	W102-K109	WNKPSKPK

Table S2: Fully tryptic, core and C-terminal ovine PrP peptides used to interrogate full-scan Q-Tof analysis of PrP^{res} preparations using MassHunter Qualitative with BioConfirm

	peptide abbreviation	sequence
No missed cleavages		
1.	R27-R40	RPKPGGGWNTGGSR
2.	Y41-R51	YPGQGSPGGNR
3.	Y52-K109	YPPQGGGGW…KPK
4.	T110-K113	TNMK
5.	H114-A136-R139	HVAGAAAAGAVVGGLGGYMLGSAMSR
6.	H114-V136-R139	HVAGAAAAGAVVGGLGGYMLGSVMSR
7.	P140-L141-R151	PLIHFGNDYEDR
8.	P140-F141-R151	PFIHFGNDYEDR
9.	Y152-R154	YYR
10.	Y152-H154-R159	YYHENMYR
11.	E155-R159	ENMYR
12.	Y160-R167	YPNQVYYR
13.	P168-R171	PVDR
14.	P168-Q171-K188	PVDQYSNQNNFVHDCepVNITVK
15.	P168-H171-K188	PVDHYSNQNNFVHDCepVNITVK
16.	Y172-K188	YSNQNNFVHDCepVNITVK
17.	Q189-K197	QHTVTTTTK
18.	G198-K207	GENFTETDIK
19.	I208-R211	IMER
20.	V212-R223	VVEQMCepITQYQR
21.	E224-R231	ESQAYYQR
22.	G232-A233	GA
Single missed cleavage		
23.	K26-R40	KRPKPGGGWNTGGSR
24.	R27-R51	RPKPGGGWNTGGSRYPGQGSPGGNR
25.	Y41-K109	YPGQGSPGGNRYPPQGGGGW…KPK
26.	Y52-K113	YPPQGGGGW…KPKTNMK
27.	T110-A136-R139	TNMKHVAGAAAAGAVVGGLGGYMLGSAMSR
28.	T110-V136-139	TNMKHVAGAAAAGAVVGGLGGYMLGSVMSR
29.	H114-A136-L141-R151	HVAGAAAAGAVVGGLGGYMLGSAMSRPLIHFGNDYEDR
30.	H114-A136-F141-R151	HVAGAAAAGAVVGGLGGYMLGSAMSRPFIHFGNDYEDR
31.	H114-V136-R151	HVAGAAAAGAVVGGLGGYMLGSVMSRPLIHFGNDYEDR
32.	P140-L141-R154	PLIHFGNDYEDRYYR
33.	P140-F141-R154	PFIHFGNDYEDRYYR
34.	P140-L141-H154-R159	PLIHFGNDYEDRYYHENMYR
35.	Y152-R159	YYRENMYR
36.	Y152-H154-R167	YYHENMYRYPNQVYYR
37.	E155-R167	ENMYRYPNQVYYR
38.	Y160-R171	YPNQVYYRPVDR
39.	Y160-Q171-K188	YPNQVYYRPVDQYSNQNNFVHDCepVNITVK
40.	Y160-H171-K188	YPNQVYYRPVDHYSNQNNFVHDCepVNITVK
41.	P168-K188	PVDRYSNQNNFVHDCepVNITVK
42.	P168-K197	PVDQYSNQNNFVHDCepVNITVKQHTVTTTTK
43.	P168-K197	PVDHYSNQNNFVHDCepVNITVKQHTVTTTTK
44.	Y172-K197	YSNQNNFVHDCepVNITVKQHTVTTTTK
45.	Q189-K207	QHTVTTTTKGENFTETDIK
46.	G198-R211	GENFTETDIKIMER
47.	I208-R223	IMERVVEQMCepITQYQR
48.	V212-R231	VVEQMCepITQYQRESQAYYQR
49.	E224-A233	ESQAYYQRGA
Two missed cleavages		
50.	K25-R40	KKRPKPGGGWNTGGSR
51.	K26-R51	KRPKPGGGWNTGGSRYPGQGSPGGNR
52.	R27-K109	RPKPGGGWNTGGSRYPGQGSPGGNRYPPQGGGGW…KPK
53.	Y41-K113	YPGQGSPGGNRYPPQGGGGW…KPKTNMK

54.	Y52-A136-R139	YPPQGGGGW…KPKTNMKHVAGAAAAGAVVGGLGGYMLGSAMSR
55.	Y52-V136-R139	YPPQGGGGW…KPKTNMKHVAGAAAAGAVVGGLGGYMLGSVMSR
56.	T110-A136-L141-R151	TNMKHVAGAAAAGAVVGGLGGYMLGSAMSRPLIHFGNDYEDR
57.	T110-A136-F141-R151	TNMKHVAGAAAAGAVVGGLGGYMLGSAMSRPFIHFGNDYEDR
58.	T110-V136-R151	TNMKHVAGAAAAGAVVGGLGGYMLGSVMSRPLIHFGNDYEDR
59.	H114-A136-L141-R154	HVAGAAAAGAVVGGLGGYMLGSAMSRPLIHFGNDYEDRYYR
60.	H114-A136-F141-R154	HVAGAAAAGAVVGGLGGYMLGSAMSRPFIHFGNDYEDRYYR
61.	H114-V136-R154	HVAGAAAAGAVVGGLGGYMLGSVMSRPLIHFGNDYEDRYYR
62.	H114-A136-L141-R159	HVAGAAAAGAVVGGLGGYMLGSAMSRPLIHFGNDYEDRYYHENMYR
63.	P140L-R159	PLIHFGNDYEDRYYRENMYR
64.	P140F-R159	PFIHFGNDYEDRYYRENMYR
65.	P140L-H154-R167	PLIHFGNDYEDRYYHENMYRYPNQVYYR
66.	Y152-H154-R167	YYRENMYRYPNQVYYR
67.	Y152-H154-Q171-K188	YYHENMYRYPNQVYYRPVDQYSNQNNFVHDCepVNITVK
68.	E155-Q171-K188	ENMYRYPNQVYYRPVDQYSNQNNFVHDCepVNITVK
69.	E155-H171-K188	ENMYRYPNQVYYRPVDHYSNQNNFVHDCepVNITVK
70.	Y160-R171-K188	YPNQVYYRPVDRYSNQNNFVHDCepVNITVK
71.	Y160-Q171-K197	YPNQVYYRPVDQYSNQNNFVHDCepVNITVKQHTVTTTTK
72.	Y160-H171-K197	YPNQVYYRPVDHYSNQNNFVHDCepVNITVKQHTVTTTTK
73.	P168-R171-K197	PVDRYSNQNNFVHDCepVNITVKQHTVTTTTK
74.	P168-Q171-K207	PVDQYSNQNNFVHDCepVNITVKQHTVTTTTKGENFTETDIK
75.	P168-H171-K207	PVDHYSNQNNFVHDCepVNITVKQHTVTTTTKGENFTETDIK
76.	Y172-K207	YSNQNNFVHDCepVNITVKQHTVTTTTKGENFTETDIK
77.	Q189-R211	QHTVTTTTKGENFTETDIKIMER
78.	G198-R223	GENFTETDIKIMERVVEQMCepITQYQR
79.	I208-R231	IMERVVEQMCepITQYQRESQAYYQR
80.	V212-A233	VVEQMCepITQYQRESQAYYQRGA
Two missed cleavages and R/P rule		
81.	Y52-A136-L141-R151	YPPQGGGGW…KPKTNMKHVAGAAAAGAVVGGLGGYMLGSAMSRPLIHFGNDYEDR
82.	Y52-A136-F141-R151	YPPQGGGGW…KPKTNMKHVAGAAAAGAVVGGLGGYMLGSAMSRPFIHFGNDYEDR
83.	Y52-V136-R151	YPPQGGGGW…KPKTNMKHVAGAAAAGAVVGGLGGYMLGSVMSRPLIHFGNDYEDR
84.	T110-A136-L141-R154	TNMKHVAGAAAAGAVVGGLGGYMLGSAMSRPLIHFGNDYEDRYYR
85.	T110-A136-F141-R154	TNMKHVAGAAAAGAVVGGLGGYMLGSAMSRPFIHFGNDYEDRYYR
86.	T110-V136-R154	TNMKHVAGAAAAGAVVGGLGGYMLGSVMSRPLIHFGNDYEDRYYR
87.	T110-A136-L141-H154-R159	TNMKHVAGAAAAGAVVGGLGGYMLGSAMSRPLIHFGNDYEDRYYHENMYR
88.	H114-A136-L141-R154-R159	HVAGAAAAGAVVGGLGGYMLGSAMSRPLIHFGNDYEDRYYRENMYR
89.	H114-V136-L141-R154-R159	HVAGAAAAGAVVGGLGGYMLGSVMSRPLIHFGNDYEDRYYRENMYR
90.	H114-F141-R154-R159	HVAGAAAAGAVVGGLGGYMLGSAMSRPFIHFGNDYEDRYYRENMYR
91.	H114-L141-H154-R167	HVAGAAAAGAVVGGLGGYMLGSAMSRPLIHFGNDYEDRYYHENMYRYPNQVYYR
92.	Y152-R154-Q171-K188	YYRENMYRYPNQVYYRPVDQYSNQNNFVHDCepVNITVK
93.	Y152-R154-H171-K188	YYRENMYRYPNQVYYRPVDHYSNQNNFVHDCepVNITVK
94.	Y152-R154-R171	YYRENMYRYPNQVYYRPVDR
95.	Y152-Q171-K197	YYHENMYRYPNQVYYRPVDQYSNQNNFVHDCepVNITVKQHTVTTTTK
96.	E155-Q171-K197	ENMYRYPNQVYYRPVDQYSNQNNFVHDCepVNITVKQHTVTTTTK
97.	E155-H171-K197	ENMYRYPNQVYYRPVDHYSNQNNFVHDCepVNITVKQHTVTTTTK
98.	Y160-R171-K197	YPNQVYYRPVDRYSNQNNFVHDCepVNITVKQHTVTTTTK
99.	Y160-Q171-K207	YPNQVYYRPVDQYSNQNNFVHDCepVNITVKQHTVTTTTKGENFTETDIK
100.	Y160-H171-K207	YPNQVYYRPVDHYSNQNNFVHDCepVNITVKQHTVTTTTKGENFTETDIK

Figure S1: Extracted Compound Chromatograms of peptides identified by BioConfirm analysis from tryptic digest of classical scrapie infected ovine brain tissue (genotype 136VV/141LL/154RR/171QQ); (A): without PK treatment, N-TAAP peptides; (B): without PK, fully tryptic peptides; (C): with PK, N-TAAP peptides; (D): with PK, fully tryptic peptides.

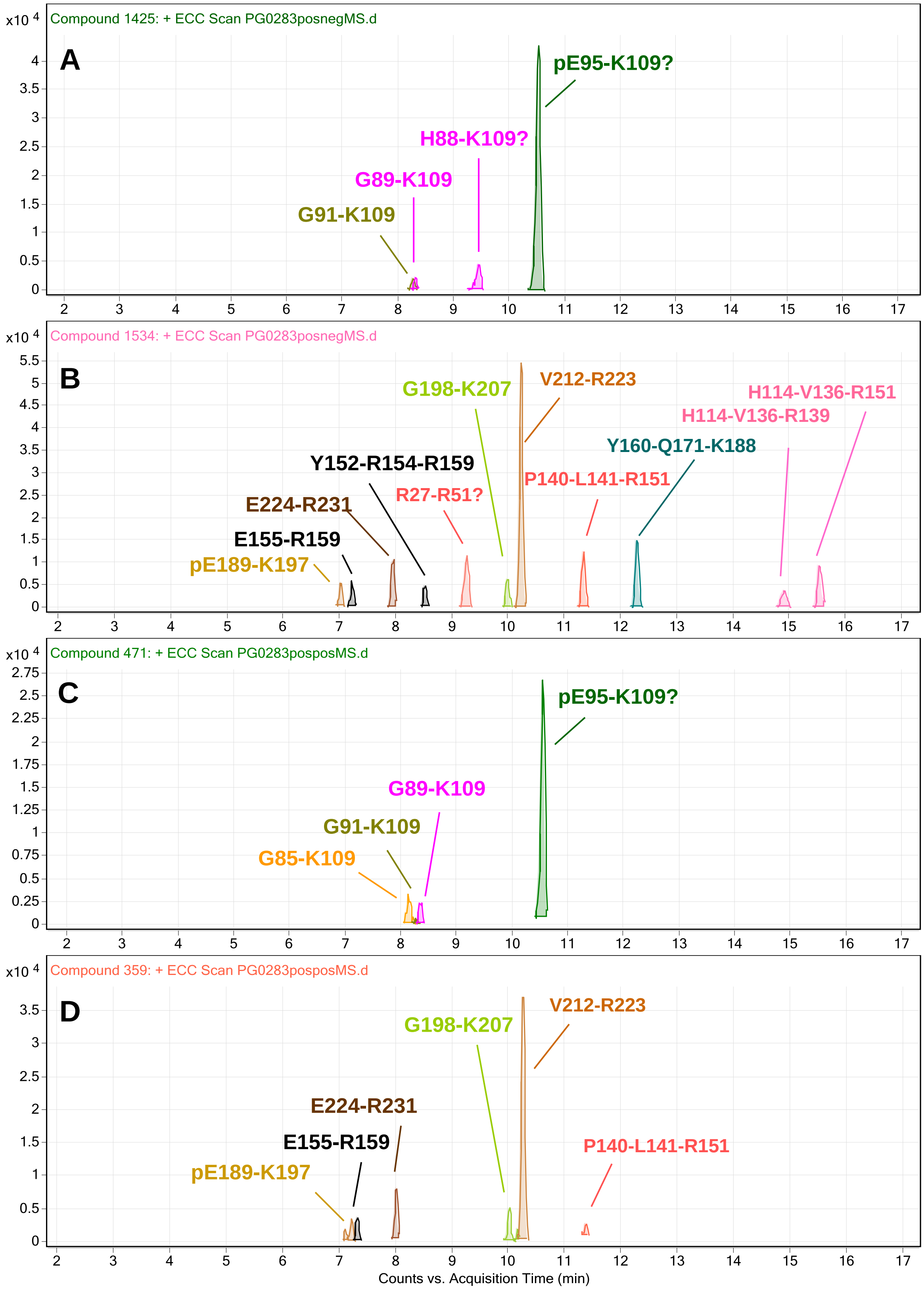

Figure S2: Extracted Compound Chromatograms of peptides identified by BioConfirm analysis from tryptic digest of CH1641 scrapie infected ovine brain tissue (genotype 136AA/141LL/154HH/171QQ): (A) without PK treatment, N-TAAP peptides; (B) without PK, fully tryptic peptides; (C) with PK, N-TAAP peptides; (D) with PK, fully tryptic peptides.

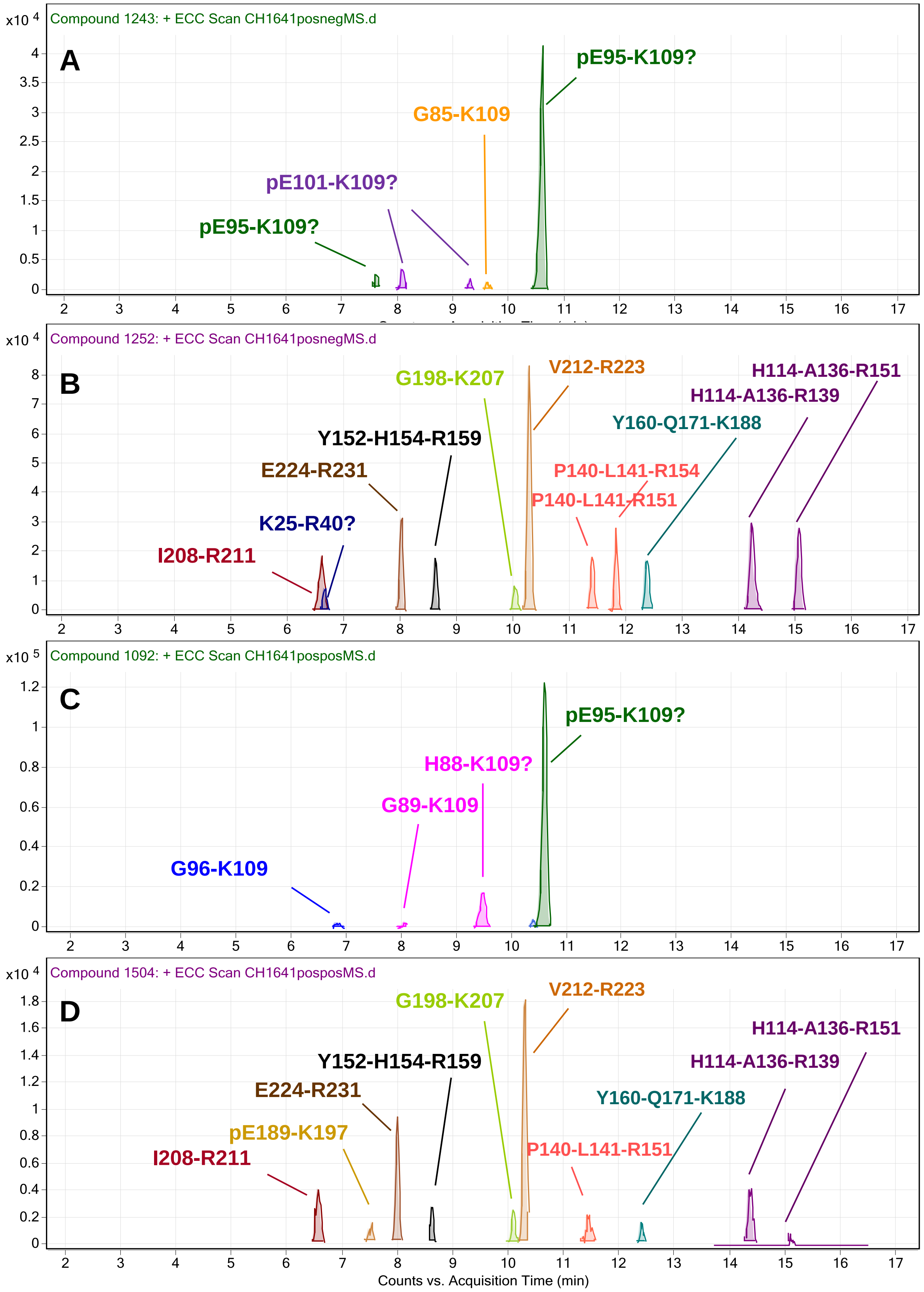

Figure S3: Extracted Compound Chromatograms of peptides identified by BioConfirm analysis from tryptic digest of BSE infected ovine brain tissue (genotype 136AA/141LL/154RR/171QQ): (A) without PK treatment, N-TAAP peptides; (B) without PK, fully tryptic peptides; (C) with PK, N-TAAP peptides; (D) with PK, fully tryptic peptides.

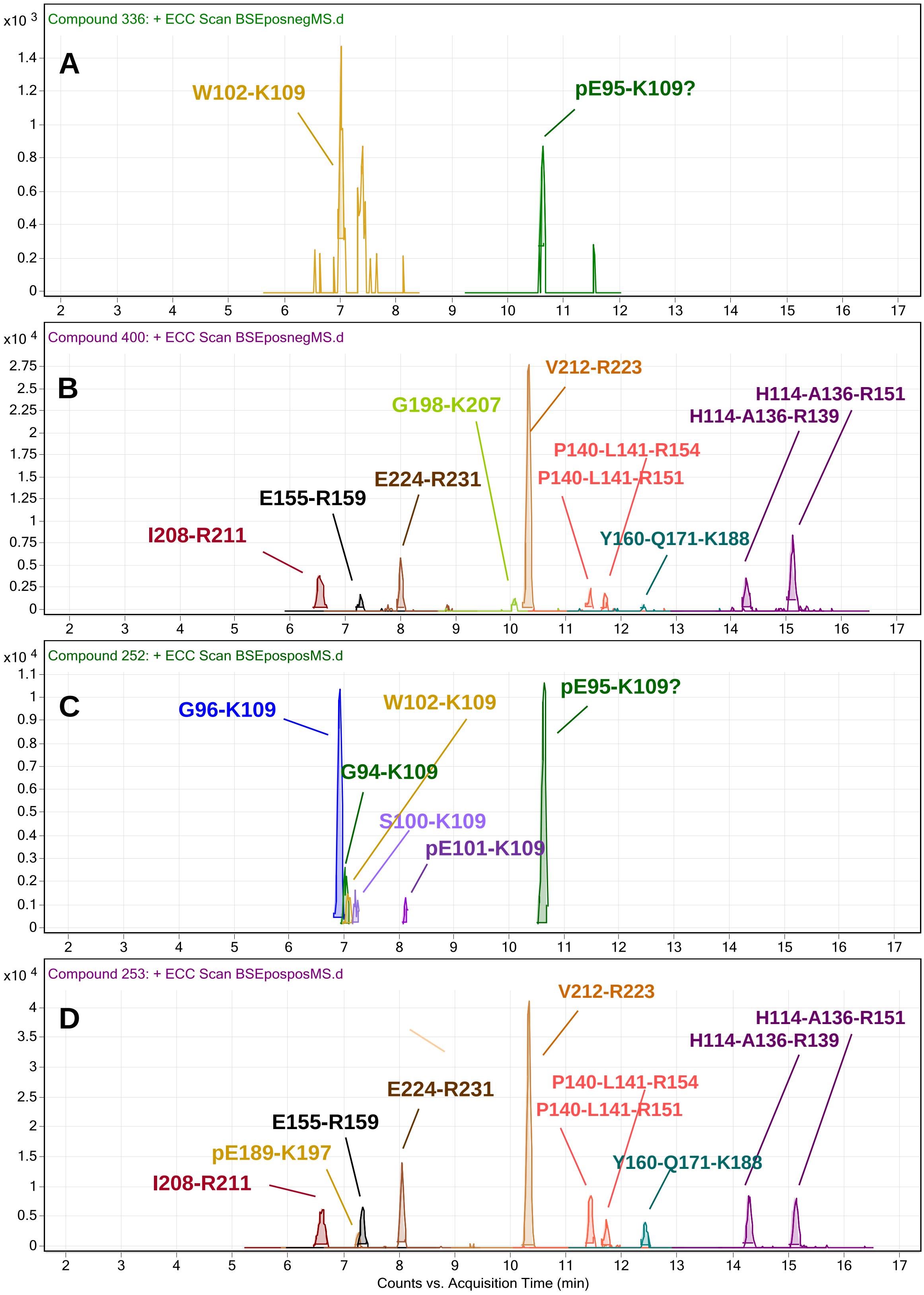

Figure S4: Extracted Compound Chromatogram of fully tryptic peptides identified by BioConfirm analysis from a tryptic digest of recombinant ovine PrP (136A/141L/154R/171Q), digested in the same buffer as used for PrP^{res} isolated from tissue. Approximately 0.4 pmol was injected. As anticipated, N-TAAP peptides could not be detected in this digest.

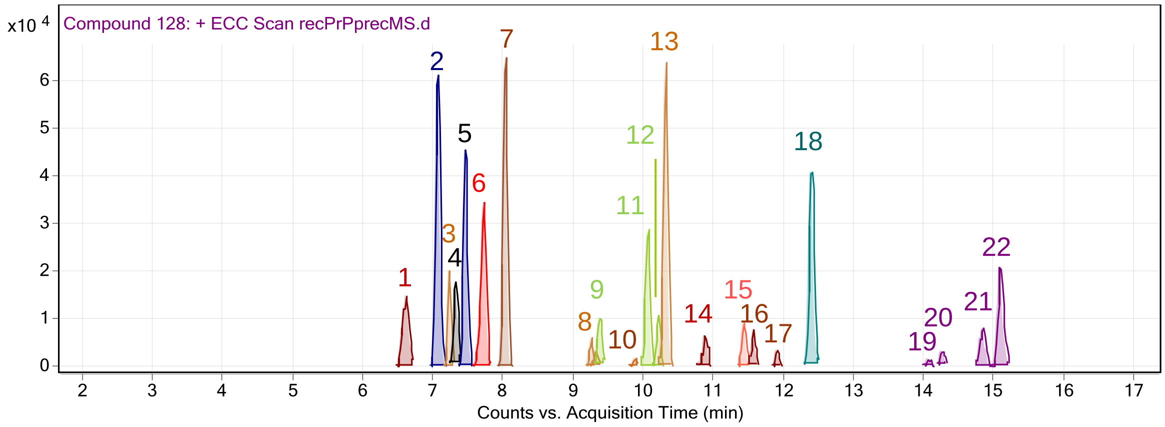

peak number	abbreviation	sequence
1	I208-R211	IMER
2	K26-R50	KRPKPGGGWNTGGSR
3	pE189-K109	pyroEHTVTTTTK
4	E155-R159	ENMYR
5	R27-R50	RPKPGGGWNTGGSR
6	Y41-R51	YPGQGSPGGNR
7	E224-R231	ESQAYYQR
8	V212-R223	VVEQMoxCepITQYQR
9	Q189-K207	QHTVTTTTKGENFTETDIK
10	Y160-R167	YPNQVYYR
11	G198-K207	GENFTETDIK
12	pE189-K207	pyroEHTVTTTTKGENFTETDIK
13	V212-R223	VVEQMCepITQYQR
14	Q189-K211	QHTVTTTTKGENFTETDIKIMER
15	P140-R151	PLIHFGNDYEDR
16	pE189-R211	pyroEHTVTTTTKGENFTETDIKIMER
17	G198-R211	GENFTETDIKIMER
18	Y160-Q171-K188	YPNQVYYRPVDQYSNQNNFVHDCepVNITVK
19	H114-R151	HVAGAAAAGAVVGGLGGYMLGSAMoxSRPLIHFGNDYEDR
20	H114-R139	HVAGAAAAGAVVGGLGGYMLGSAMSR
21	H114-R154	HVAGAAAAGAVVGGLGGYMLGSAMSRPLIHFGNDYEDRYYR
22	H114-R151	HVAGAAAAGAVVGGLGGYMLGSAMSRPLIHFGNDYEDR

Table S3: mSRM transitions used for ovine PrP N-TAAP and tryptic peptides

	Peptide abbreviation	Sequence	Precursor → product ion(s) (m/z)
1.	G77-K109	GQPHGGGWGQPHGGGGWGQGGSHSQWNKPSKPK	556.5 → 630.5<br>556.5 → 626.9
2.	G81-K109	GGGWGQPHGGGGWGQGGSHSQWNKPSKPK	583.8 → 700.9<br>583.8 → 640.2
3.	G85-K109	GQPHGGGGWGQGGSHSQWNKPSKPK	512.3 → 713.2<br>512.3 → 593.8
4.	Q86-K109	QPHGGGGWGQGGSHSQWNKPSKPK	500.6 → 575.5<br>500.6 → 497.3<br>417.2 → 513.3
5.	pE86-K109	pEPHGGGGWGQGGSHSQWNKPSKPK	497.3 → 575.0<br>497.3 → 513.3<br>497.3 → 494.0
6.	P87-K109	PHGGGGWGQGGSHSQWNKPSKPK	475.4 → 575.2<br>475.4 → 513.5<br>396.3 → 513.4
7.	H88-K109	HGGGGWGQGGSHSQWNKPSKPK	455.9 → 575.3<br>455.9 → 550.5<br>455.9 → 513.5
8.	G89-K109	GGGGWGQGGSHSQWNKPSKPK	535.3 → 675.2<br>535.3 → 575.3
9.	G90-K109	GGGWGQGGSHSQWNKPSKPK	520.8 → 656.1<br>520.8 → 575.3
10.	G91-K109	GGWGQGGSHSQWNKPSKPK	506.6 → 637.0<br>506.6 → 575.3
11.	G92-K109	GWGQGGSHSQWNKPSKPK	492.4 → 769.5<br>492.4 → 575.3
12.	W93-K109	WGQGGSHSQWNKPSKPK	478.0 → 575.3<br>478.0 → 473.8
13.	G94-K109	GQGGSHSQWNKPSKPK	431.7 → 550.5<br>431.7 → 513.3
14.	Q95-K109	QGGSHSQWNKPSKPK	417.2 → 550.4<br>417.2 → 412.3<br>334.0 → 330.6
15.	pE95-K109	pEGGSHSQWNKPSKPK	413.0 → 513.4<br>413.0 → 494.5<br>413.0 → 408.7
16.	G96-K109	GGSHSQWNKPSKPK	385.4 → 494.5<br>385.4 → 475.3
17.	G97-K109	GSHSQWNKPSKPK	371.2 → 556.4<br>371.2 → 493.0<br>371.2 → 366.7
18.	S98-K109	SHSQWNKPSKPK	357.0 → 556.4<br>357.0 → 493.0<br>357.0 → 400.0
19.	H99-K109	HSQWNKPSKPK	335.2 → 556.4<br>335.2 → 399.9<br>335.2 → 330.9
20.	S100-K109	SQWNKPSKPK	400.7 → 548.4<br>400.7 → 492.9

21.	Q101-K109	QWNKPSKPK	371.5 → 556.6<br>371.5 → 365.9<br>278.9 → 365.7
22.	pE101-K109	pEWNKPSKPK	365.9 → 556.3<br>365.9 → 459.3<br>365.9 → 360.3
23.	W102-K109	WNKPSKPK	328.9 → 556.2<br>328.9 → 323.1
24.	Y41-R51	YPGQGSPGGNR	545.4 → 829.4<br>545.4 → 644.3<br>545.4 → 463.9
25.	Y41-Hyp47-R51	YPGQGSHypGGNR	553.4 → 845.5<br>553.4 → 660.4<br>553.4 → 471.9
26.	H114-A136-R139	HVAGAAAAGAVVGGLGGYMLGSAMSR	777.7 → 862.5<br>777.7 → 608.2
27.	H114-V136-R139	HVAGAAAAGAVVGGLGGYMLGSVMSR	787.6 → 862.5<br>787.6 → 636.2
28.	H114-A136-L141-R151	HVAGAAAAGAVVGGLGGYMLGSAMSRPLIHFGNDYEDR	758.9 → 972.1<br>758.9 → 939.1<br>758.9 → 863.3
29.	H114-V136-L141-R151	HVAGAAAAGAVVGGLGGYMLGSVMSRPLIHFGNDYEDR	764.5 → 981.3<br>764.5 → 948.4<br>764.5 → 872.7
30.	H114-A136-L141-R154	HVAGAAAAGAVVGGLGGYMLGSAMSRPLIHFGNDYEDRYYR	855.4 → 1099.9<br>855.4 → 1024.1<br>712.9 → 825.1
31.	H114-V136-L141-R154	HVAGAAAAGAVVGGLGGYMLGSVMSRPLIHFGNDYEDRYYR	717.7 → 859.9<br>717.7 → 832.2
32.	P140-L141-R151	PLIHFGNDYEDR	492.6 → 689.8<br>492.6 → 576.6
33.	P140-F141-R151	PFIHFGNDYEDR	504.1 → 706.9<br>504.1 → 576.7
34.	P140-L141-H154-R159	PLIHFGNDYEDRYYHENMYR	527.6 → 676.0<br>527.6 → 578.4<br>527.6 → 110.0
35.	P140-F141-R154	PFIHFGNDYEDRYYR	499.0 → 676.0<br>499.0 → 545.7<br>499.0 → 279.6
36.	P140-L141-R154	PLIHFGNDYEDRYYR	490.6 → 749.6<br>490.6 → 676.1<br>490.6 → 545.9
37.	Y152-H154-R159	YYHENMYR	392.7 → 506.7<br>392.7 → 425.2
38.	Y152-R154-R159	YYRENMYR	399.1 → 516.4<br>399.1 → 434.9
39.	E155-R159	ENMYR	357.0 → 469.1<br>357.0 → 315.3
40.	Y160-R167	YPNQVYYR	551.8 → 842.4<br>551.8 → 470.2
41.	Y160-R171	YPNQVYYRPVDR	524.2 → 704.1<br>524.2 → 655.5<br>524.2 → 469.8

42.	Y160-H171-K188	YPNQVYYRPVDHYSNQNNFVHDCepVNITVK	727.6 → 868.5<br>727.6 → 695.0<br>606.5 → 579.3
43.	Y160-Q171-K188	YPNQVYYRPVDQYSNQNNFVHDCepVNITVK	725.8 → 762.7<br>725.8 → 722.4
44.	P168-H171-K188	PVDHYSNQNNFVHDCepVNITVK	510.6 → 567.3<br>510.6 → 449.1
45.	P168-Q171-K188	PVDQYSNQNNFVHDCepVNITVK	635.8 → 804.5<br>635.8 → 440.1
46.	Y172-K188	YSNQNNFVHDCepVNITVK	525.7 → 617.3<br>525.7 → 567.3
47.	Q189-K197	QHTVTTTTK	339.5 → 450.4<br>339.5 → 327.6<br>339.5 → 321.8
48.	pE189-K197	pEHTVTTTTK	500.3 → 751.4<br>500.3 → 551.3<br>500.3 → 491.7
49.	Q189-K207	QHTVTTTTKGENFTETDIK	538.5 → 649.0<br>538.5 → 605.0<br>538.5 → 534.0<br>538.5 → 529.0<br>538.5 → 525.0
50.	pE189-K207	pEHTVTTTTKGENFTETDIK	712.0 → 943.5<br>712.0 → 843.8<br>712.0 → 706.3
51.	Q189-R211	QHTVTTTTKGENFTETDIKIMER	670.8 → 666.7<br>536.9 → 705.5<br>536.9 → 533.5
52.	pE189-R211	pEHTVTTTTKGENFTETDIKIMER	666.6 → 806.0<br>666.6 → 739.2<br>666.6 → 662.3
53.	G198-K207	GENFTETDIK	577.3 → 967.4<br>577.3 → 706.3
54.	I208-R211	IMER	274.5 → 435.1<br>274.5 → 304.3
55.	V212-R223	VVEQMCepITQYQR	535.0 → 752.0<br>535.0 → 702.9
56.	E224-R231	ESQAYYQR	522.9 → 629.3<br>522.9 → 466.1

Table S4: Limits of Detection (LoDs) and Lower Limits of Quantification (LLoQs) determined for individual PrP peptides

	peptide	LoD (fmol/µL)	LloQ (fmol/µl)
1.	Y41-R51	0.05	0.05
2.	Y41-Hyp47-R51	0.2	0.2
3.	G77-K109	10	10
4.	G81-K109	2	2
5.	G85-K109	1	1
6.	Q86-K109	2	2
7.	pE86-K109	1	5
8.	P87-K109	2	50
9.	H88-K109	0.5	0.5
10.	G89-K109	0.5	5
11.	G90-K109	0.2	0.2
12.	G91-K109	1	1
13.	G92-K109	0.5	1
14.	W93-K109	0.2	0.5
15.	G94-K109	0.2	0.2
16.	Q95-K109	0.1	0.5
17.	pE95-K109	1	1
18.	G96-K109	0.02	0.2
19.	G97-K109	0.5	0.5
20.	S98-K109	50	50
21.	H99-K109	0.1	0.2
22.	S100-K109	0.1	0.2
23.	Q101-K109	0.05	10
24.	pE101-K109	0.02	0.02
25.	W102-K109	0.05	0.1
26.	H114-A136-R139	5	5
27.	H114-V136-R139	2	5
28.	H114-A136 R151	1	5
29.	H114-V136-R151	2	2
30.	H114-A136-R154	0.1	0.5
31.	H114-V136-R154	5	100
32.	P140-L141-R151	0.2	0.5
33.	P140-F141-R151	0.1	0.1
34.	P140-L141-R154	1	1
35.	P140-F141-R154	0.05	1
36.	P140-L141-H154-R159	0.5	1
37.	Y152-H154-R159	0.1	0.1
38.	Y152-R154-R159	1	1
39.	E155-R159	5	5
40.	Y160-R167	0.05	0.05
41.	Y160-R171	0.05	0.05
42.	Y160-H171-K188	0.2	0.5
43.	Y160-Q171-K188	2	2
44.	P168-H171-K188	0.1	1
45.	P168-Q171-K188	0.5	1
46.	Y172-K188	0.1	1
47.	Q189-K197	0.05	>100
48.	pE189-K197	0.05	0.1
49.	Q189-K207	0.5	0.5
50.	pE189-K207	0.05	0.05
51.	Q189-R211	1	1
52.	pE189-R211	5	5
53.	G198-N200-K207	10	10
54.	I208-R211	0.05	0.05
55.	V212-R223	5	5
56.	E224-R231	0.1	0.2

Figure S5: mSRM Chromatograms of peptides identified by BioConfirm analysis from the same tryptic digests of classical scrapie infected ovine brain tissue as in Figure S1: (A) without PK treatment, N-TAAP peptides; (B) without PK, fully tryptic peptides; (C) with PK, N-TAAP peptides; (D) with PK, fully tryptic peptides. Interference: high abundance transition also found in pE101-K109, not accompanied by other pE101-K109 transitions and with different retention time.

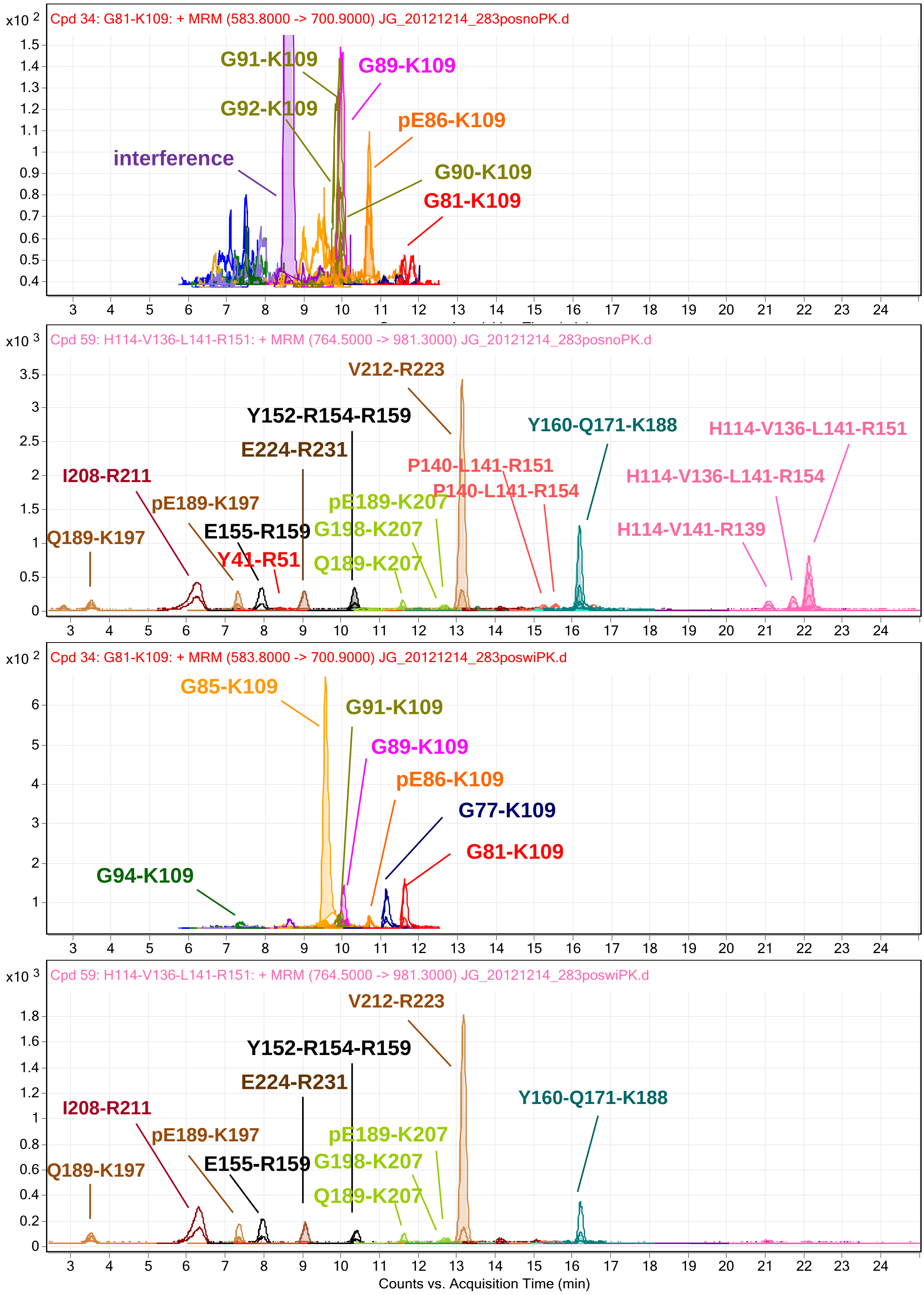

Figure S6: Extracted Compound Chromatograms of peptides identified by BioConfirm analysis from the same tryptic digests of CH1641 scrapie infected ovine brain tissue as in Figure S2: (A) without PK treatment, N-TAAP peptides; (B) without PK, fully tryptic peptides; (C) with PK, N-TAAP peptides; (D) with PK, fully tryptic peptides.

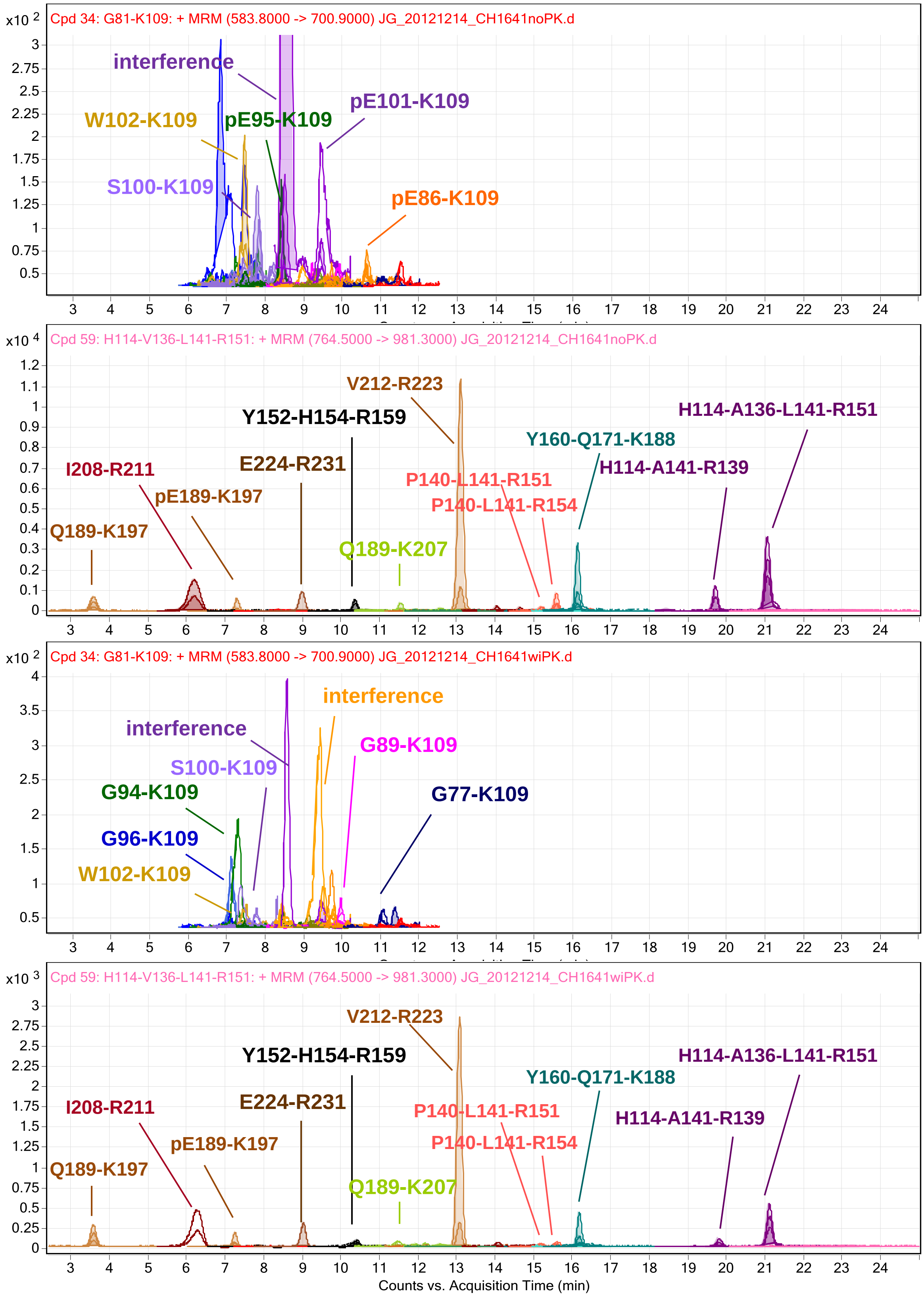

Figure S7: Extracted Compound Chromatograms of peptides identified by BioConfirm analysis from the same tryptic digests of BSE infected ovine brain tissue as in Figure S3: (A): without PK treatment, N-TAAP peptides; (B) without PK, fully tryptic peptides; (C) with PK, N-TAAP peptides; (D) with PK, fully tryptic peptides. N-TAAP peptides could not be identified by BioConfirm analysis in the BSE sample without PK treatment.

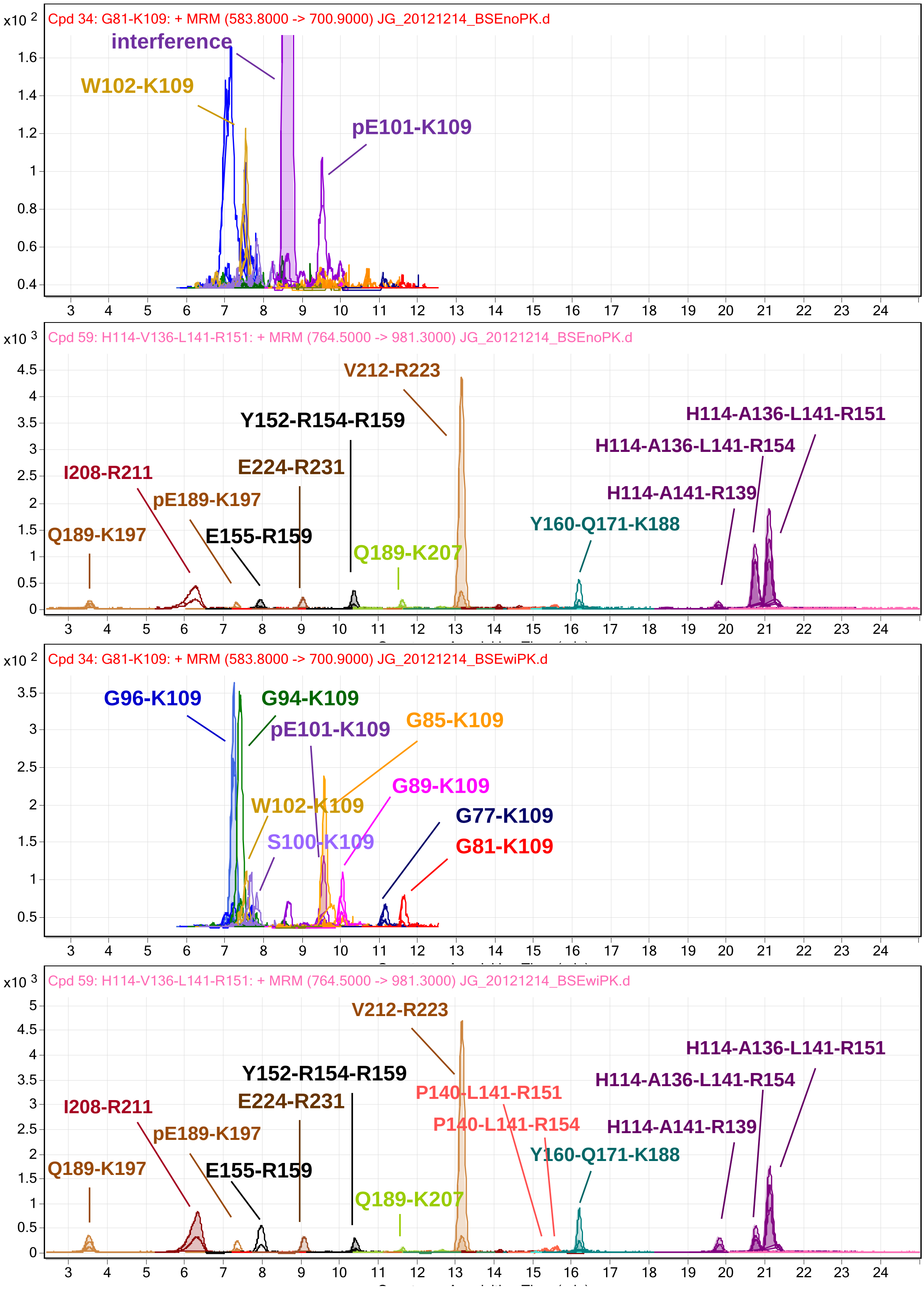

Figure S8: Tryptic peptide profiles from ovine TSEs following quantitative mSRM analysis, corresponding to the samples of which the N-TAAPs are shown in Figure 4. Graphs show calculated concentrations (±SD) of N-TAAP peptides determined by chip-HPLC SRM mass spectrometry of digest preparations from the brain stem of individual animals. (A) Classical scrapie: VRQ/VRQ Swaledale, neutered male, homebred; (B) Experimental CH1641 scrapie: AHQ/AHQ Cheviot, neutered male; (C) Experimental BSE: ARQ/ARQ Romney, Asterisks are used point out the peptides with pyroglutamate N-termini.

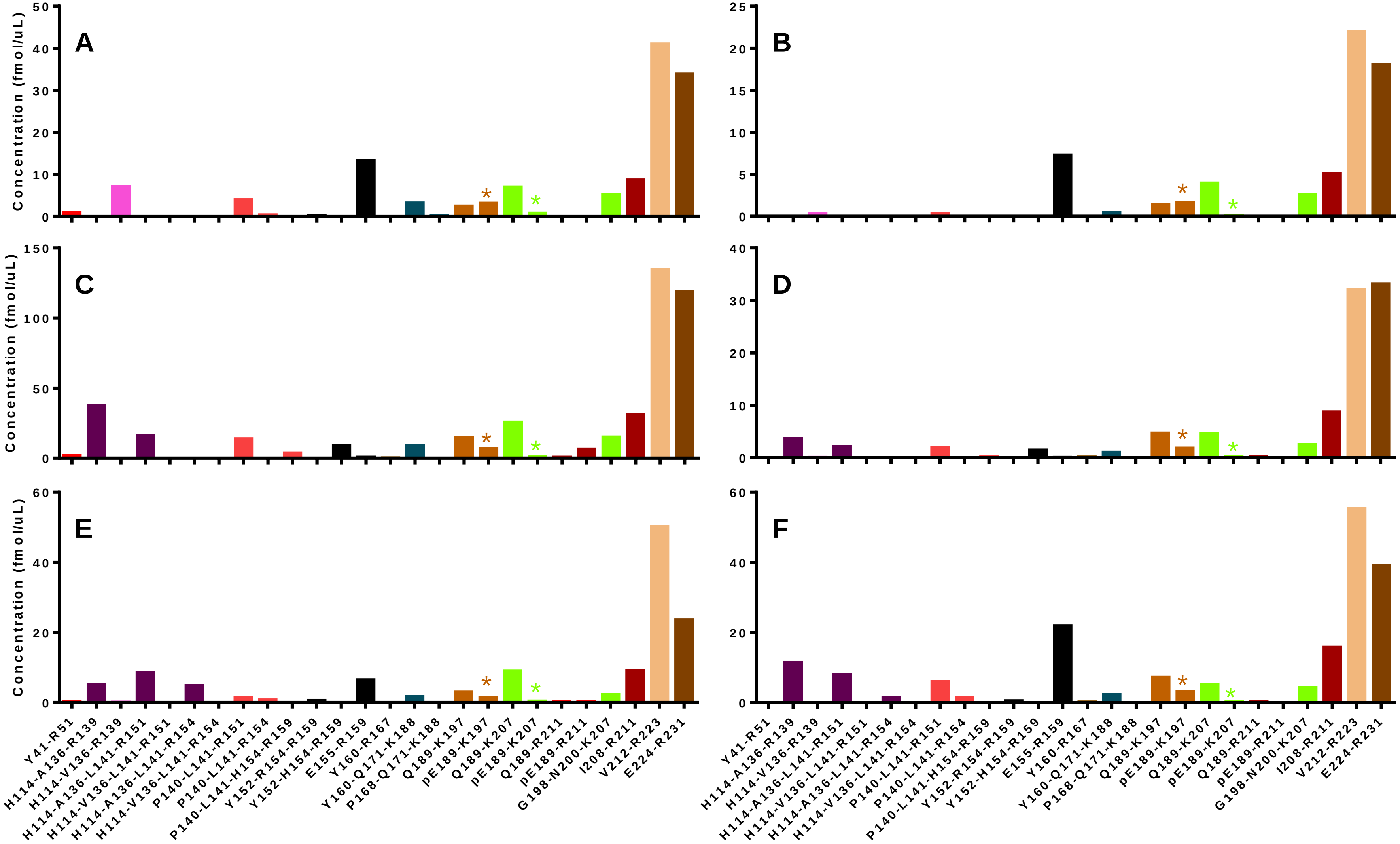

Figure S9: Tryptic peptide profiles from classical scrapie cases the N-TAAPs of which are shown in Figure 5. Graphs show calculated concentrations (±SD) of N-TAAP peptides determined by chip-HPLC SRM mass spectrometry of digest preparations from the brain stem of individual animals, obtained by processing 2x1.75 ml of 10% homogenate. (A) ARQ/ARQ Swaledale, neutered male, homebred; (B) VRQ/VRQ Swaledale, female, homebred; (C) ARH/VRQ Texel, female, purchased into flock from farm A; (D) ARQ/VRQ white-faced Dartmoor, female, homebred (E, F) ARQ/ARQ Vendeen, female, purchased into flock from farm B.

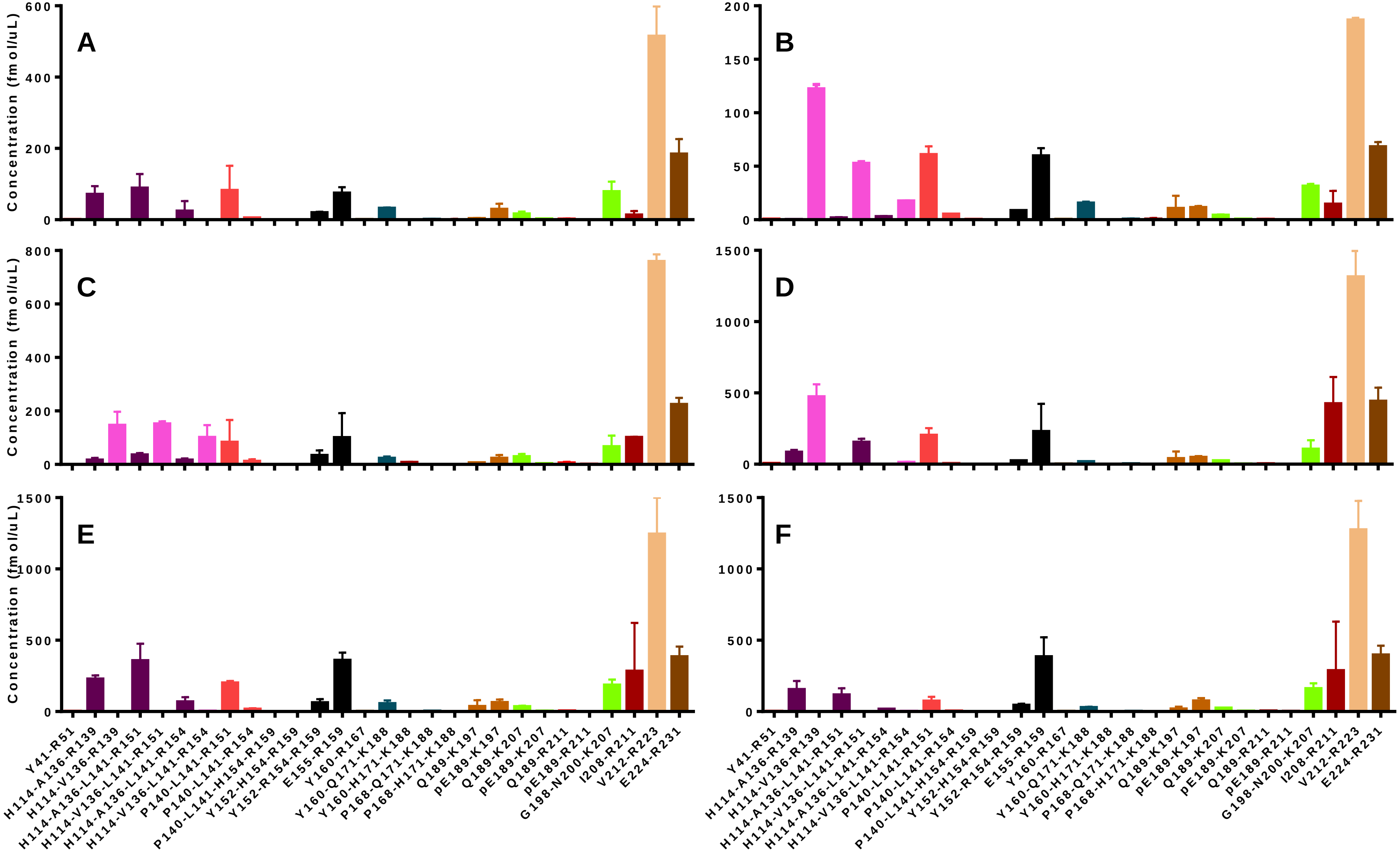

Supplement: Supplementary file 1 [file Presentation1.PDF]
